# Zinc and Copper Have the Greatest Relative Importance for River Macroinvertebrate Richness at a National Scale

**DOI:** 10.1021/acs.est.4c06849

**Published:** 2025-02-17

**Authors:** Andrew C. Johnson, Dinara Sadykova, Yueming Qu, Virginie D.J. Keller, Nuria Bachiller-Jareno, Monika D. Jürgens, Michael Eastman, François Edwards, Clarissa Rizzo, Peter M. Scarlett, John P. Sumpter

**Affiliations:** ! UK Centre for Ecology and Hydrology, Wallingford OX10 8BB, U.K.; @ University of Exeter, Mathematics and Statistics, Harrison Building, Streatham Campus, North Park Road, Exeter EX4 4QF, U.K.; # Met Office, FitzRoy Road, Exeter EX1 3PB, U.K.; $ APEM Ltd, Riverview A17 Embankment Business Park Vale Road, Stockport SK4 3GN, U.K.; % Wallingford Hydrosolutions, Howbery Business Park, Wallingford OX10 8BA, U.K.; & Department of Life Sciences, Brunel University London, Heinz Wolff Building, Kingston Lane, Uxbridge UB8 3PH, U.K.

**Keywords:** river, freshwater invertebrates, statistical modeling, chemical stressors, habitat and geographic conditions

## Abstract

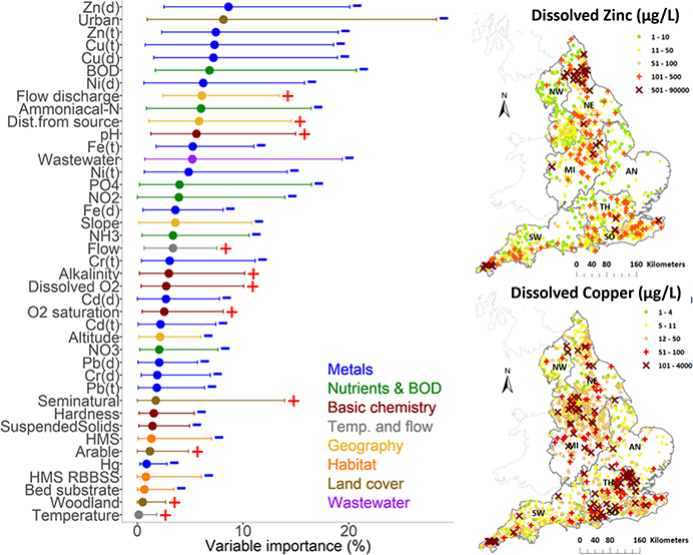

It is important to discover what change led to the improvement in European macroinvertebrate biodiversity in the period from 1990−2000s and what prevents further desirable gains from taking place today. A 30-year data set from 1,457 macroinvertebrate monitoring sites spread across England, with 65,032 discrete observations was combined with 41 chemical, physical, habitat, and geographic variables. This data set was analyzed using generalized linear mixed-effect models and generalized additive mixed models. To include all combinations of the variables required to address each question, required over 20,000 model runs. It was found that no variables were more consistently and strongly associated with the overall family richness than Zn and Cu. Zn and Cu led both for the era of large gains in richness up to 2005 and also in the later period of 2006–2018 when few further gains were made.

## Abstract 2

Across England’s rivers over the past 30 years from 41 different chemical, physical, geographic, and habitat variables, none were more closely associated with freshwater invertebrate diversity than Zn and Cu.

## Introduction

River ecosystems are particularly vulnerable to chemical pollution because of their connectivity and intimate association with human population centers and agriculture. Many observers have noticed an improvement in freshwater invertebrate biodiversity, notably in richness (*i.e.*, the number of taxa), across Europe and North America, that began in the late 1980s to early 1990s.^1−10^ The similarity in timing of the improvement across European freshwater biodiversity is striking and implies a decline in one or more universal stressor(s). This European increase in diversity was followed by a plateauing in family richness at a suboptimal level, from the mid to late 2000 period onward.^2,8,9,11^ Meanwhile, the so-called sensitive aquatic insects Ephemeroptera, Plecoptera, and Trichoptera (EPT) have continued to increase in richness, unabated, since around 1990.^2,9,11^

The big question is what has driven this intriguing increase in overall macroinvertebrate diversity since the 1990s and, perhaps more importantly, what has prevented further desirable increases in richness from occurring since the late 2000 period?^8,11^ This significant increase in overall richness cannot be attributed to the arrival of alien species.^8,9^ It is reasonable to expect that the decline in gross organic pollution, ammonia, and nutrients, with the introduction of the European Urban Wastewater Directive (to be complied with by 1998) will have played an important role in helping many lowland and urban rivers.^12,13^ A popular theory to explain why no further significant improvements in richness have taken place since the mid-2000 period is that new pollutants, such as organic micropollutants, have taken their place.^2,8^ Previous studies, which have included statistical methods to identify associations with invertebrate richness, have flagged up temperature, insecticides, flow, livestock, forestry, urban land cover, cropland, nutrient levels, gross organic pollution, and river physical habitat^1,4,5,8,14−19^ as all playing greater or lesser roles. However, these studies are limited by either the short duration of their study period, the low numbers of sites, or, more importantly, the low numbers of variables examined together.

This study used data from 1,457 macroinvertebrate monitoring sites spread across every English region, from upland to lowland, from seminatural areas to urban catchments, and from small to large rivers, with a mean of 21 sampling years (sampling years per site range from 5 to 38 during 1972–2018).^9^ They provided 65,032 observations that were integrated with 41 different colocated variables in space and (for many) in time, including physical, chemical, geographic, and habitat factors.^20^ The major questions addressed in this study were:

- To rank which variables were most closely associated with macroinvertebrate family richness using the entire data set (temporal and spatial).
- To assess whether this ranking of variable importance to macroinvertebrates differed between the historic period of significant richness increase (1989–2005) and the subsequent more recent period of relative richness stability (2006–2018).
- To assess whether a spatial component might influence the ranking of variable importance for macroinvertebrate richness.
- To identify if thresholds could be identified where the variables of greatest interest influenced macroinvertebrate richness.
- To examine whether the subgroup of Ephemeroptera, Plecoptera, and Trichoptera (EPT) family richness differed in terms of responses to variables compared to overall family richness.

The statistical analysis was conducted using generalized linear mixed-effect models with Template Model Builder and natural splines (GLMM-TMB-NS), which incorporated natural splines to capture nonlinear effects, random influences and accounted for seasonality and the temporal structure using the Ornstein–Uhlenbeck model.^21,22^

## Methodology

### Collecting an Integrated Macroinvertebrate and Related Variables Data Set

The collection of 1,457 macroinvertebrate sites used in the statistical analyses was colocated with the widest possible range of explanatory environmental variables (most importantly, nearby chemical monitoring sites).^20^ The macroinvertebrate monitoring data (BIOSYS database), chemical data (WIMS), and many physical/habitat covariables are publicly available for England, thanks to their collection and curation by the Environment Agency. The macroinvertebrate sites were selected on the basis of their longevity of records and representativeness of all geographic regions of England. The 41 variables included chemicals, upstream land cover, habitat scores, river flow, air temperature, wastewater exposure, and physical factors such as altitude, slope, and distance from the source of the river (Table 1). An explanation of each variable and how the information was collected is provided in the Supporting Information. The full data set that was brought together to carry out the statistical analysis can be found at ref. (23).

**Table 1:** List of Environmental Variables Used in Statistical Analysis^a^

Physical and habitat variables	General water quality	Metal concentrations
5 percentile low flow (described as flow)^b^	NO_3_ (maximum)	Fe(d) as the mean
Flow discharge (size of river at sampling point)	NO_2_ (maximum)	Fe(t) as the mean
Altitude	pH (mean)	Pb(d) as the mean
Distance from source (described as dist. from source)	PO_4_ (mean)	Pb(t) as the mean
Slope	% O_2_ saturation (minimum)	Hg(t) as the mean
Habitat modification score (described as HMS)	Dissolved O_2_ (minimum)	Ni(d) as the mean
Bed resectioning (described as HMS RBBSS)	Suspended solids (mean)	Ni(t) as the mean
Bed substrate	Alkalinity (mean)	Zn(d) as the mean
Proportion upstream urban land cover (described as urban)	Ammoniacal-N (maximum)	Zn(t) as the mean
Proportion upstream seminatural land cover (described as seminatural)	BOD (mean)	Cd(d) as the mean
Proportion upstream cropland land cover (described as arable)	Hardness (mean)	Cd(t) as the mean
Proportion upstream woodland land cover (described as woodland)	Air temperature (mean)	Cr(d) as the mean
Local mean wastewater exposure (described as wastewater)		Cr(t) as the mean
		Cu(d) as the mean
		Cu(t) as the mean

a The solutes use concentration data such as mg/L. Land cover is the proportion of the catchment upstream attributed to one of the four classes. Mean wastewater exposure is a modeled % wastewater contribution to natural flow. The non-geographic variables used a summary statistic considered appropriate (mean, minimum, or maximum) are taken from the preceding 6 months of records from the date of the macroinvertebrate sample. A full description of each variable and its origins is provided in the Supporting Information.

b One of several flow statistics tested. This low flow metric had slightly more relevance (see Supporting Information).

### Examination of the GLMM-TMB-NS (and GAMM) Modeling Process

The generalized linear mixed-effect model using Template Model Builder with natural splines (GLMM-TMB-NS) was selected as the most suitable statistical technique to apply to this data set and to address the study’s main objectives. Explained deviance within the GLMM-TMB-NS framework allowed us to rank these variables by their influence on richness, addressing our primary objective. By applying GLMM-TMB-NS consistently across different temporal subsets, we examined shifts in variable importance over time, while spatial subsets were used to explore regional differences, addressing additional objectives. Finally, to confirm the robustness of these results, we employed generalized additive mixed models (GAMMs) as a supplementary check. However, the GAMM approach proved to be more complex to run, and so corroboration efforts were limited to the first major question of the study.

A GLMM-TMB-NS run incorporated two chemical variables, one habitat variable, one physical variable, and one land variable, representing a total of five variables of interest. These models also included a time covariance structure, random effects, and seasonal effects. The number of observations varied across the models, each involving different sets of variables, ranging from 2,893 to 45,562, with a mean value of 11,783. We consider the combination of chemical, physical, habitat, and land cover present in each model to be an appropriate method of testing the strength of each variable.

Here, we let *y*_*tRS*_ denote either the family richness (FR) or EPT richness observed at a specific time point *t*, within a particular region denoted by *R*, and at a specific site indicated by *S*. We assume that *y*_*tRS*_ follows a negative binomial distribution with linear parametrization,^24^ which is similar to the “quasi-Poisson” parametrization (because it matches the linear mean-variance relationship assumed by quasi-Poisson models). Further, we assumed that
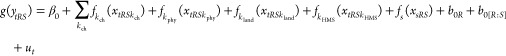
1

where *g*(·) is the log link function, β_0_ is the intercept of the model, , , and  correspond to the chemical, physical, land, and habitat modification score (HMS) variables, respectively, also given at a time point *t* and at a site *S* within a region *R*. *x*_*sRS*_ is a seasonal variable (given as months). The effects of the chemical, physical, land, HMS, and seasonal variables were modeled as smooth functions f_*_(·) with natural splines.

The degree of freedom for the natural splines was selected based on the Akaike Information Criterion (AIC) for each variable. We limited the maximum degrees of freedom to five to prevent the model from overfitting the data. We observed that a maximum of 5 degrees of freedom was frequently chosen across most models, indicating that the data required a relatively flexible nonlinear modeling approach. The  term represents sums computed over different combinations of chemical variables. In our analysis, we considered all possible combinations of two chemical covariates (additionally, we conducted an extra analysis testing up to six chemical variables to assess the percentage of deviance explained by the full model (1)). There are several parameters in the data set where two versions of roughly the same thing were reported, such as the dissolved and total concentration of a chemical such as ammoniacal nitrogen and ammonia, or dissolved oxygen and oxygen saturation. Because the data set does not always include both versions of these parameters for a particular sampling occasion, both parameters were included in the analysis, but never together in the same model run. Models that included other variables (chemical, physical, land, HMS, and seasonal) with high correlation coefficients (>0.7) were also excluded, to mitigate issues associated with multicollinearity. The strategy of considering all possible combinations of both chemical and nonchemical variables, in clusters of five in the GLMM-TMB-NS, was driven by the presence of an extensive number of missing values, particularly among the chemical variables. This approach, where each model contains at least two chemicals, a habitat variable, a physical variable, and a land cover type, allowed for the inclusion in the modeling of each site where at least two chemical variables coexisted. The number of observations available per environmental variable is shown in Table S1. The sometimes-high number of missing variables meant that an imputation approach was not valid (for more detailed information, see Supporting Information). To review all possible combinations of all variables in clusters of 5 for the 41 variables, required 20,796 separate model runs. This was after eliminating some models because of highly correlated variables, duplicated variables (total and dissolved concentration of the same variable), or when the variables had a similar origin (such as ammoniacal nitrogen and ammonia).

All numerical explanatory variables were scaled and centered to enhance comparability, and all models were constructed with identical complexity.

The notation *b*_0*R*_ denotes the random intercept that accounts for region variability, while *b*_0[*R*:*S*]_ represents the nested random effects of sites within regions, accounting for the variability of the sites within a region (in other words, the intercept varying among sites within regions). Both random effects (*b*_0*R*_ and *b*_0[*R*:*S*]_) are assumed to follow a Gaussian distribution and are integrated out using the Laplace approximation.^21^ Because of convergence issues encountered when employing nested random effects within the generalized linear mixed-effect model using the Template Model Builder with natural splines (GLMM-TMB-NS) framework, all presented results are based on the models with random effects at the regional level (with a 100% convergence rate). However, a comparative analysis of outcomes obtained from models that successfully converged with nested random effects (approximately half of the models) against a subset of these models, using regional-level random effects only, demonstrated a remarkable similarity in terms of variable importance, showing the same parameters as the top variables.

Although the generalized additive mixed model (GAMM) (the second alternative approach) framework demonstrated satisfactory convergence rates when employing nested random effects, difficulties emerged when incorporating a number of smoothed explanatory variables, leading to convergence failures. Consequently, we retained the nested structure for the GAMM framework but reduced the number of explanatory variables to two chemical variables plus one of the following: either the HMS variable, the physical variable, or the land variable.

Finally, *u*_*t*_ is the Ornstein–Uhlenbeck covariance structure that incorporates temporal autocorrelation between consecutive observations. This covariance structure is especially useful in modeling time series data, where the observations are dependent on their previous values. Additionally, this covariance structure accounts for irregular time points.

Modeling was performed using the glmmTMB function in R^21^ (glmmTMB is an abbreviation for “generalized linear mixed-effect models with the Template Model Builder”). The Template Model Builder approach was selected based on its ability to offer more efficient and faster computational performance, when compared to alternative algorithms. This is achieved by employing automatic differentiation along with the Laplace approximation. GAMM modeling was performed using the gamm4 function in R.

Having fitted the models described above to the data, the overall explained deviance by any full model was calculated using the following formula:^25^
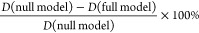


The notation “*D*(model)″ denotes the deviance of the model, where the specific model is defined within the parentheses. “Null model” refers to the null model, which only contains an intercept term and random effects. “Full model” refers to the full model, which incorporates all the variables (5 variables for GLMM-TMB-NS and only 3 for GAMM), as described above.

To quantify the extent to which each environmental variable contributes to explaining the deviance of a full model, defined here as variable importance, the following formula^25^ was applied:
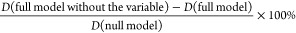


The term “full model without the variable” denotes the full model with the exclusion of the variable under consideration.

It should be noted that the deviance explained by each variable, as well as by any full model, was calculated as the percentage improvement. This calculation approach, which focuses on the relative change in deviance rather than absolute values, ensures comparability of variable importance across different data sets and models, even when null model deviances vary.

The variable importance (as the contribution to explaining the deviance) was computed for each (chemical and nonchemical) environmental variable within each full model. The resulting values indicating the relative importance of each environmental variable are presented as mean values across all models, with the range reflecting the minimum and maximum values across all models. To provide a simple explanation of how the GLMM-TMB-NS process works (in this case handling five variables at a time), as an example, if one started with the variables Zn, ammonia, habitat score, flow, and arable land, the model would collect data from all macroinvertebrate sites where all five of these variables are present together (20,592 observations in this particular case). Then, it would calculate how much of the variability of the macroinvertebrate family richness was explained by these five variables combined. It would then repeat this exercise but eliminate one variable at a time, for example, Zn, and it would generate a new relationship value with family richness. The difference would reveal the importance that Zn held in that original mixture in explaining the deviance. This process is then repeated for each variable in the group of five. Thus, it is possible to gauge the individual importance of each variable in that original mix of five. This is just one model run. The next model run might include Zn, Hg, riverbed resectioning, temperature, and woodland as its variables (with 5,650 observations in that case), and the process would be repeated until all possible combinations of five were completed.

### Why GLMM-TMB-NS Was Chosen for the Statistical Examination of This Data Set

The GLMM-TMB-NS framework was selected because of its suitability for analyzing data with complex structures, such as sites nested within regions, which account for both site-level and regional-level variations, thus reducing the risks of biased estimates. The Ornstein–Uhlenbeck covariance structure accommodates temporal autocorrelation, accounting for the high similarity in measurements obtained in close temporal proximity, while also allowing the use of irregular time points. Natural splines account for complex nonlinear relationships in the data. The employed modeling methodology enables the incorporation and adjustment for seasonal variation. In addition, a negative binomial distribution with linear parametrization was employed to account for the presence of overdispersion in the counts of macroinvertebrate family richness and EPT-family richness.

Overall, the GLMM-TMB-NS approach was thought to be particularly suitable to address the challenges associated with this type of data set (such as complex relationships, temporal autocorrelation, irregular measurements, nested spatial structure, and seasonality). The generalized linear mixed model approach has frequently been used in ecology when trying to tease apart relationships between diversity and a range of variables.^26−29^

### Applying a Classification and Regression Tree (CART) Approach to Explore the Threshold Value for Decision Making for the Key Variables

CART is a decision tree that learns from data inputs in machine learning. Data are partitioned along the predictor axes into subsets with homogeneous values of the dependent variable. The criterion is set as the split value that improves the relative error by a predetermined value, which, in this case, was a complexity parameter of 0.05. The specific value of the parameter at the split acts as a threshold. In this case, the threshold provides insights into critical values of the parameter that have the greatest impact on the family richness value. Rather than constructing a tree using several variables here, we included all sites and dates containing macroinvertebrate observations with either dissolved Zn or Cu only. The choice of focusing only on Zn and Cu came from the outcome of the GLMM-TMB-NS statistical analysis of relative variable importance.

## Results

### Trends in Macroinvertebrate Richness from 1972 to 2018

**Figure 1:**
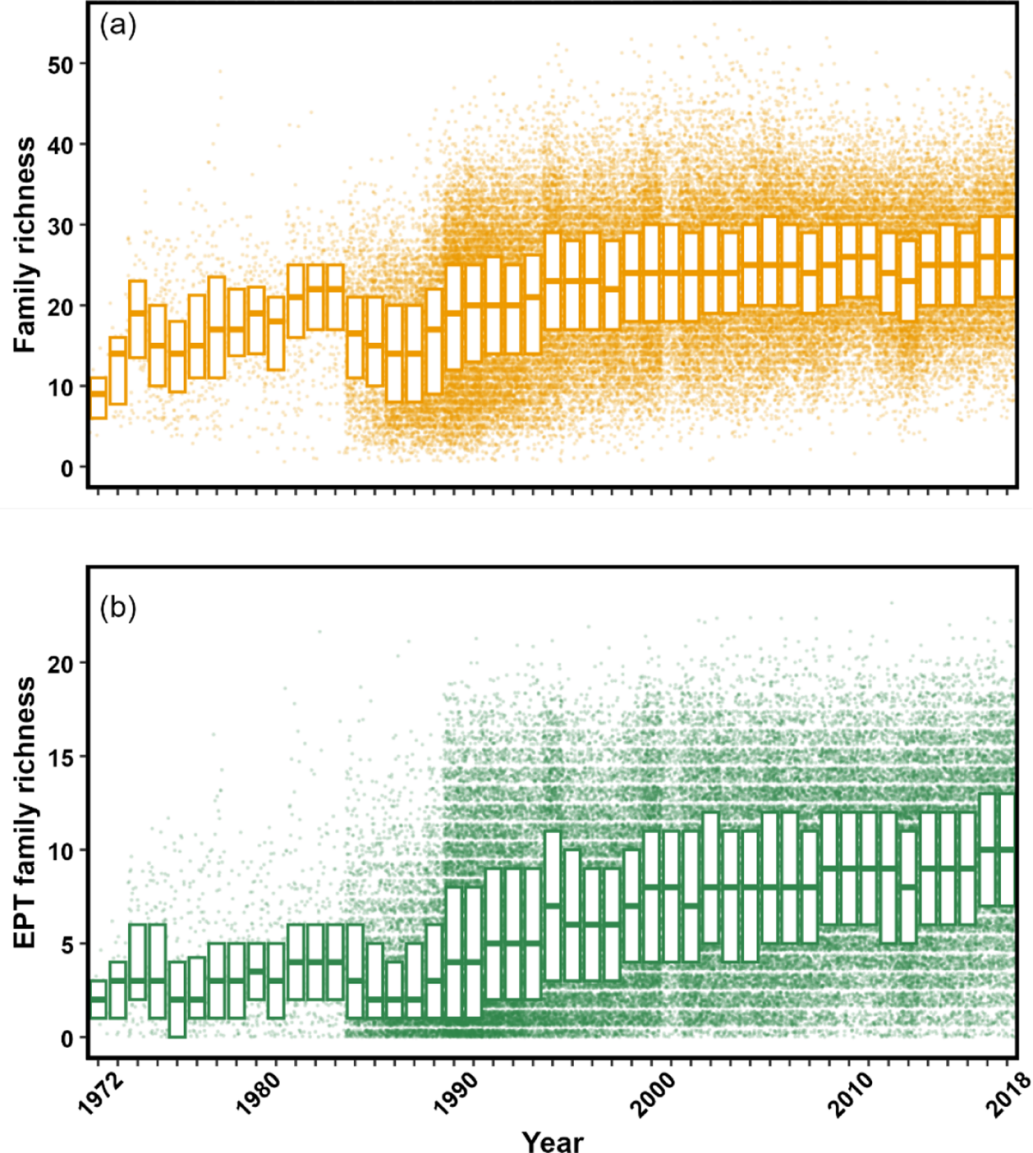
Change in macroinvertebrate richness of the total family number of the community (a) and the family number from EPT orders (b) over time for 1,519 observation sites and all observations across England (showing median, 25th and 75th percentiles).

Usually, a macroinvertebrate monitoring site was visited twice a year in spring and autumn. The mean number of visits to a monitoring site to record the macroinvertebrates present was 89 (range = 18–127). The trends for overall family richness and for the sensitive EPT family richness demonstrate increases over time (Figure 1). The overall family richness appears to have plateaued somewhere in the mid-2000 period (Figure 1a), but with some continuing increase in the EPT family richness (Figure 1b). Further details on the relative significance of these changes in richness can be found in Qu et al. (2023).^9^

### Identifying the Environmental Variables Most Closely Associated with Macroinvertebrate Richness

**Figure 2:**
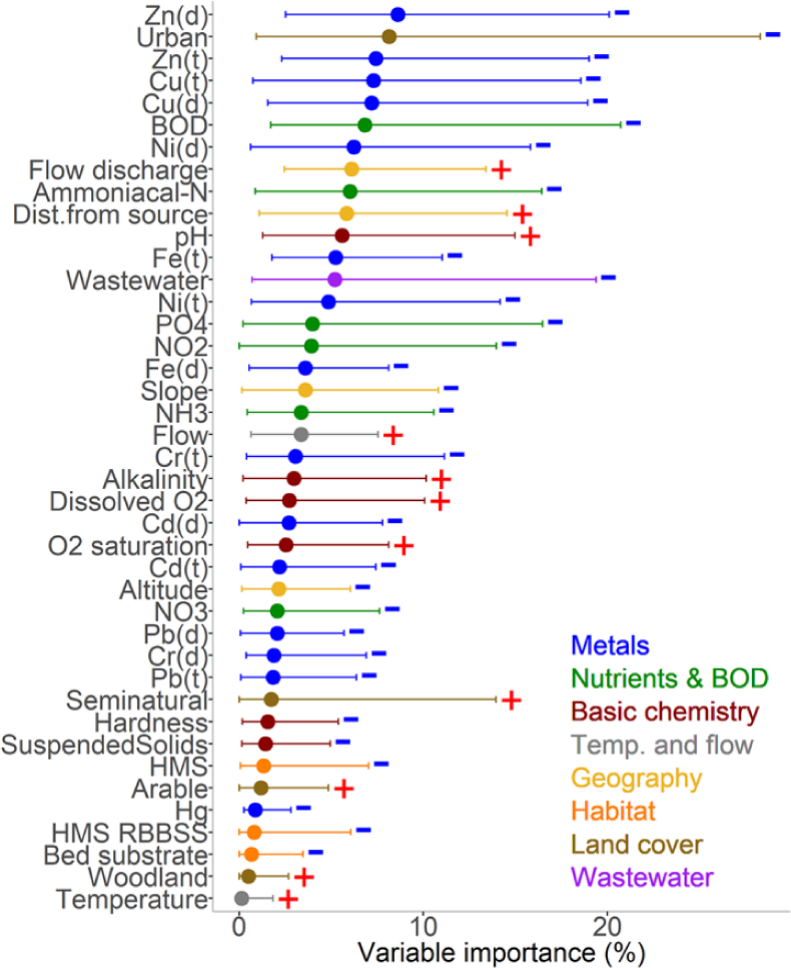
Relative importance of environmental variables to overall macroinvertebrate family richness for England (based on 1,457 sites and 65,032 observations) from 1972 to 2018 from a GLMM-TMB-NS statistical model, presented as a percentage of explained deviance by each variable. Dots represent mean values across the models. Lines indicate the range of explained deviations, with the left and right parts corresponding to minimum and maximum percentages for each variable, respectively. Positive or negative relationships are denoted by plus and minus signs. The metric for the solutes is the mean, minimum, or maximum value for the preceding 6 months from the macroinvertebrate sample (Table 1). Note that for the metals, there are usually two entries: dissolved (filtered through a 0.45 μm membrane) and total. HMS refers to habitat modification score, and HMS_RBBSS to a score specifically for modification of the river channel and bed substrate as a habitat related score for the riverbed substrate. Flow here is the 5th percentile low flow metric.

The outcome, summarized as mean importance values across all models and showing the range from minimum to maximum importance values across the models, allowed the identification of relative importance with respect to macroinvertebrate family richness (Figure 2).

The degree of explained deviance by a model containing 5 variables could reach up to 50%. As a separate test, it was found that with 8 variables (either 6 chemical variables and any 2 nonchemical variables, or 5 chemical variables and any 3 nonchemical variables), the percentage of explained deviance could reach up to 73%, although this result was derived from models with a reduced number of observations (generally fewer than 3,500 observations). That a selection from these 41 variables could account for so much of the overall macroinvertebrate richness variability is an indication that many of the key variables have been included.

The result of the model analysis indicates that all 41 variables play a role in influencing overall macroinvertebrate family richness, but that some are much more important than others (Figure 2). The chemical variables most closely associated with richness for England as a whole were Zn and Cu. This does not mean they were the most important in every river, just that they were more important, more often, than the others. It is crucial to note that there were instances where models revealed a limited influence of Zn and Cu. For example, Figures S1 and S2 show the influence of the proportion of wastewater in the river flow at the monitoring site on the importance of the different variables. The sites with higher wastewater levels revealed a higher importance for BOD and ammonia/ammonium compared with Zn and Cu. Upstream urban land cover also features very highly as an important factor for overall family richness. While these relationships, marked as positive or negative, are based on the entire data set, they may vary within smaller subsets of data. When the analysis was repeated with the different statistical approach of generalized additive mixed models (see Figure S3), a very similar result was found with Zn and Cu coming to the fore.

### Do the Variables Which Influence Macroinvertebrate Richness Change with Time?

**Figure 3:**
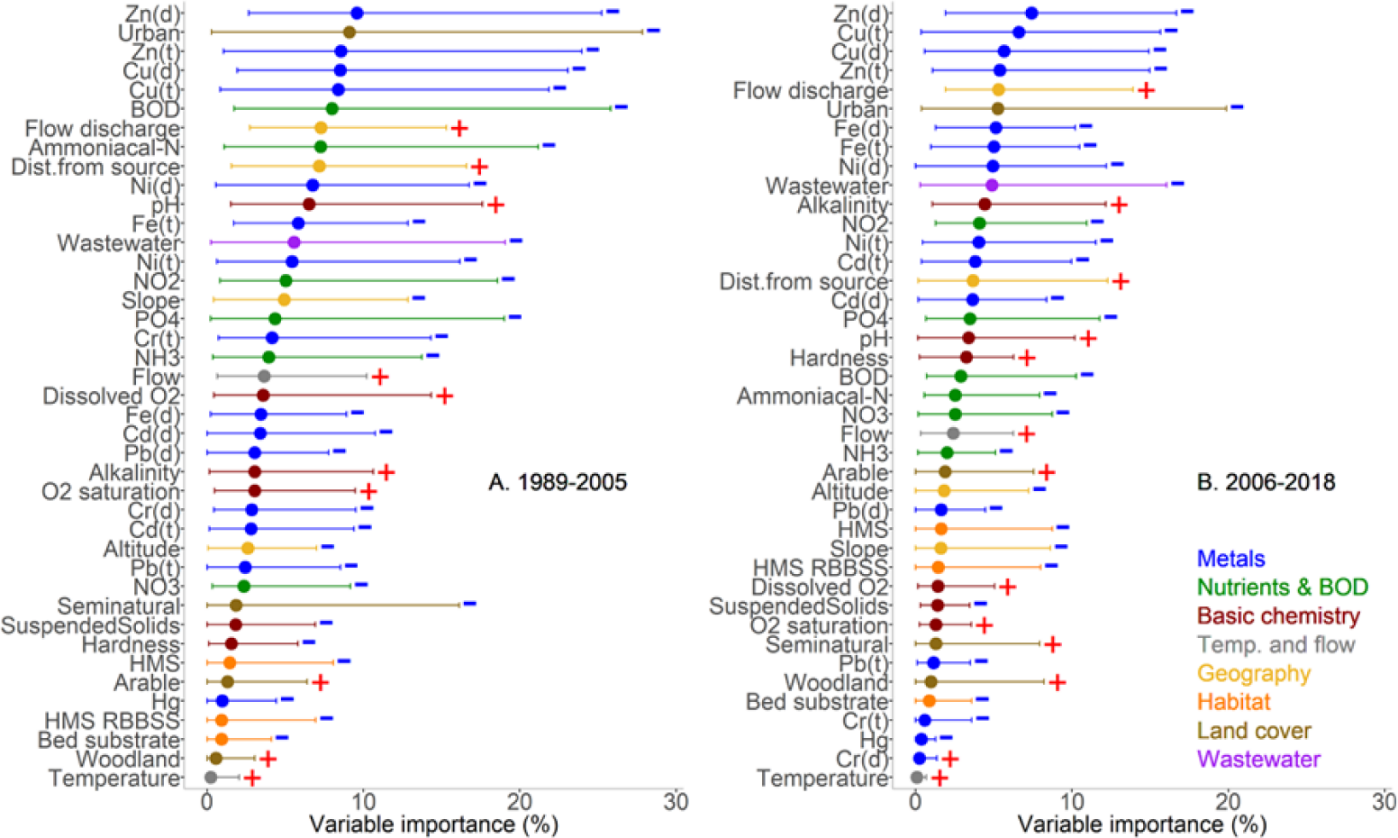
Relative importance of variables from the GLMM-TMB-NS statistical model to macroinvertebrate family richness for England presented as a percentage of explained deviance by each variable. Results based on 1,457 sites for the periods 1989–2005 (36,699 observations) (A; left) and 2006–2018 (23,440 observations) (B; right). Dots represent mean values across models. Lines indicate the range of explained deviance, with the left and right parts corresponding to minimum and maximum percentages for each variable, respectively. Positive or negative relationships are denoted by plus and minus signs.

There have been broad improvements in major water quality determinands, such as the concentration of metals and nutrients in England over the past 30 years, but the biggest reductions occurred in the 1989–2000 period.^12^ Thus, it is possible that historic decreases in concentrations of the basic water quality chemicals were the driving factors for macroinvertebrate diversity in the 1989–2005 period, but that other chemicals or unknown factors played a more important role in the most recent time period (2006–2018). However, if the data are divided into two for these different periods and the models rerun, it is found that Zn and Cu lead in importance for associations with richness for both the historic and recent periods (Figure 3). In contrast, ammoniacal-N and BOD dropped down the ranking of important variables in the more recent 2006–2018 period compared to their prominence in the earlier period. Although somewhat fewer observations were available for the latter period (there are 36,699 observations for the years 1989–2005 and 23,440 observations for the years 2006–2018), it remains a considerable data set. Similarly, the sites that were sampled in the first and second periods were broadly the same and without any regional bias.^9^

The analysis shows that the proportion of explained deviance in the later 2006–2018 period is less than in the 1989–2005 period for the 41 variables. This could reflect both the stabilization of family richness due to improved environmental conditions, leading to less variability, as well as the continued influence of unaccounted-for factors, such as microorganic pollutants playing a greater role in the more recent time period; yet wastewater exposure, which is a proxy for all and any domestic origin organic chemicals, did not gain importance in the more recent period.

### Do the Variables Associated with Macroinvertebrate Richness Change with Latitude?

In a previous analysis of national trends in macroinvertebrates,^9^ it was apparent that there was only one part of England where overall family richness was declining following earlier gains, and that was in the North (above 54.5° latitude). The selected division by latitude had simply been chosen to divide England into four equal parts, from north to south. A subsequent review of the chemical trends in the northern latitude showed that Zn stood out as having declined to 40 μg/L by 2005 before steadily rebounding to 200 μg/L by 2017 (see Figure S4). Historically, the North of England was a major Zn mining area.^30^ These areas remain important sources of metal pollution.^31^ Conceivably, greater rainfall extremes post-2007 associated with climate change might have mobilized Zn from old mine workings and spoil heaps.^32^ There are historic Zn mines in other parts of the country, notably in Cornwall where high Zn levels are also experienced (Figure 5). However, these other Zn hotspots tend to be more dotted around the coast and not in the center of the region, as in the north. These observations supported the prominence of Zn as influencing macroinvertebrate diversity but, at the same time, they raised the question as to whether the national statistical modeling results were unduly influenced by a powerful relationship perhaps unique to the North of England. Therefore, the statistical analysis was rerun, excluding monitoring sites above 54.5° latitude (where many former Zn mines exist). The statistical analysis still found an important association with Zn from the Midlands southward, where levels below 50 μg/L are more common, in other words, excluding the Northern Region with its rich Zn-mining heritage (Figure S6). This demonstrated that a relatively strong association with Zn remains for macroinvertebrate diversity throughout the country, even in regions without a mining history.

### Does the EPT Subgroup of Aquatic Insects Respond to a Different Set of Variables than Those Identified for Overall Family Richness?

**Figure 4:**
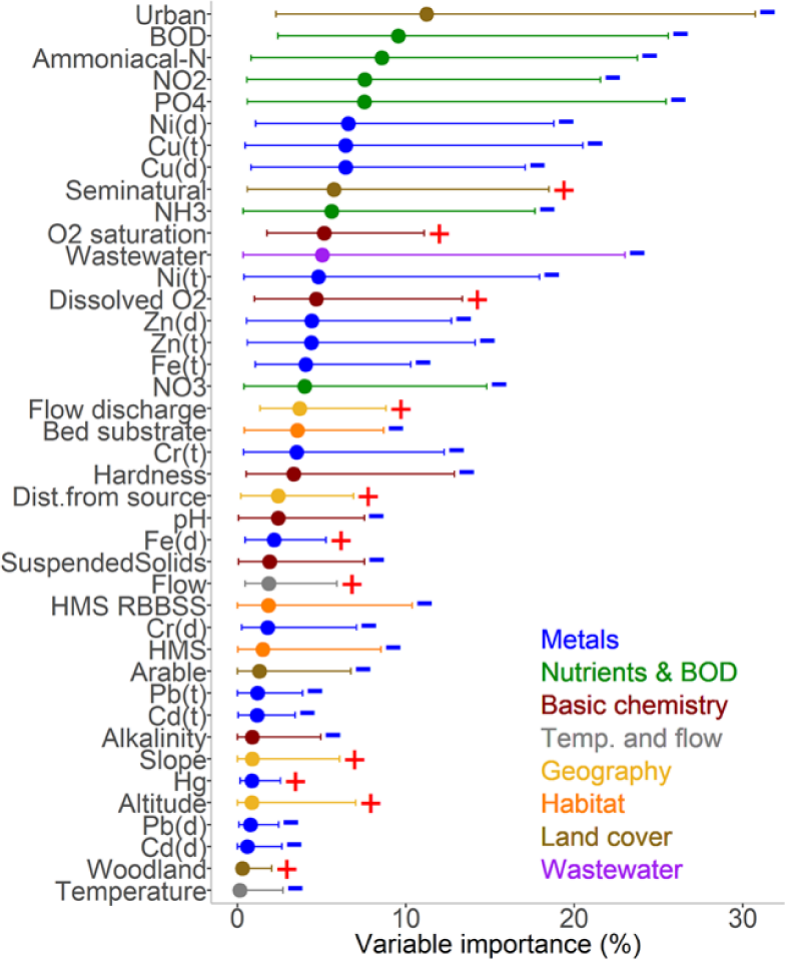
Relative importance of variables to EPT family richness for England based on 1,457 sites and 65,032 observations from the GLMM-TMB-NS statistical model, presented as a percentage of explained deviance by each variable. Dots represent mean values across models. Lines indicate the range of explained deviance, with the left and right parts corresponding to minimum and maximum percentages for each variable, respectively. Positive or negative relationships are denoted by plus and minus signs.

Unlike overall family richness (Figure 1a), which on average has plateaued at a level below the reference condition,^9^ EPT family richness (Figure 1b), which features strongly in the average score per taxon, ASPT metric, has not slowed but continued its upward trend.^2,9^ For the EPT group, the GLMM-TMB-NS statistical analysis identifies BOD, ammonia, nitrite, and phosphate as the most important associations rather than Zn and Cu (Figure 4). That EPT richness continues to increase would imply that BOD, ammonia, nitrite, and phosphate have declined to levels which are now less limiting nationally than was previously the case.

### The Relationship between Zn and Cu Levels, Ecotoxicity Thresholds, and Family Richness Outcomes

**Figure 5:**
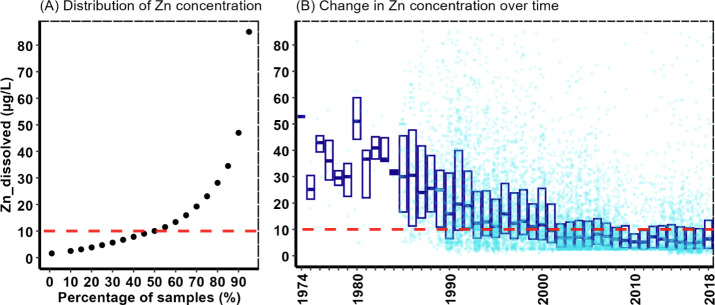
Comparison of national Zn river concentrations with a plausible Zn toxicity threshold. The graph on the left (A) shows the proportion of all observations exceeding a 10 μg/L Zn toxicity threshold. The graph on the right (B) shows the trend in Zn concentrations over time. There are 8,468 Zn-dissolved observations with the boxplots showing the median along with the 25th and 75th percentile values for each year. Note that the plots focus on the central 95% of the data to improve visualization, as the data set is highly skewed with some extreme values, but all data were included in the modeling and analysis.

**Figure 6:**
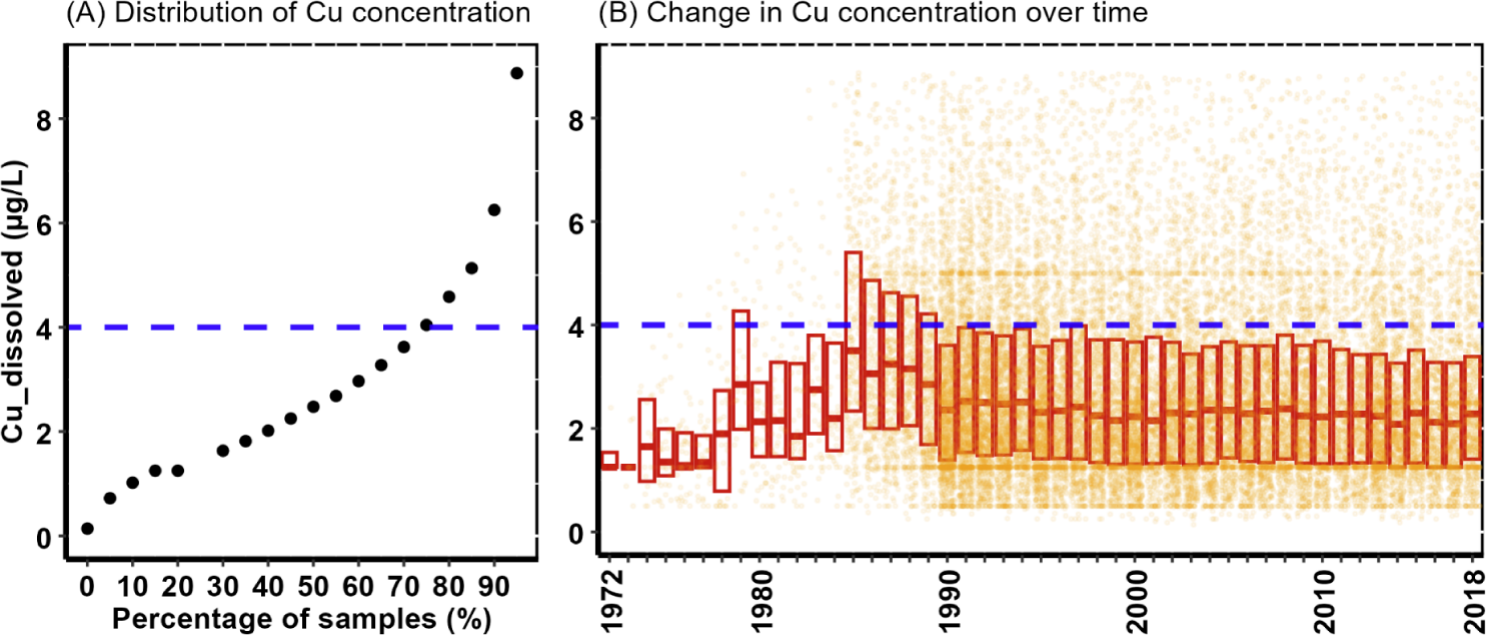
Comparison of national Cu river concentrations with a plausible Cu toxicity threshold. The graph on the left (A) shows the proportion of all observations exceeding a 4 μg/L Cu toxicity threshold. The graph on the right (B) shows the trend in Cu concentrations over time. There are 25,420 Cu observations with the boxplots showing the median along with the 25th and 75th percentile values for each year. Note that the plots focus on the central 95% of the data to improve visualization, as the data set is highly skewed with some extreme values, but all data were included in the modeling and analysis.

Plotting all the Zn and Cu results over time shows a declining trend in the 1980–1990s before reaching relative stability from the 2000 period onward (Figures 5 and 6). Metal behavior in water is complicated with multiple factors combining to enhance or reduce toxicity.^33^ In this exercise, as an illustration, we identified Zn and Cu levels that, on the basis of the ecotoxicity literature, could plausibly cause harm to invertebrates under some water chemistry conditions; the rationale for the concentrations chosen is given in the Supporting Information. For Zn, 50% of the values measured in English rivers were found to exceed an estimated lowest ecotoxicity level of 10 μg/L (Figure 5). With Cu, 30% of the values found in rivers exceeded the estimated lowest ecotoxicity level of 4 μg/L (Figure 6). These apparently high levels of risk for Zn and Cu in English rivers have been previously reported with respect to risks from other organic pollutants.^34^ The wide geographic distribution of sites across the country experiencing Zn or Cu levels above these thresholds is shown in Figure S5.

**Figure 7:**
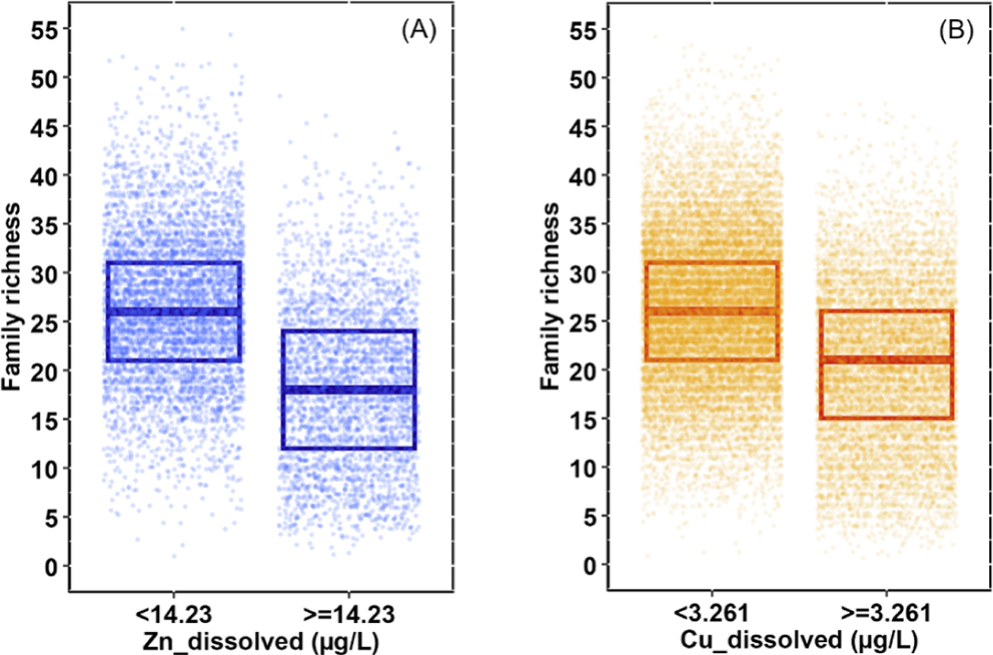
Identifying a threshold Zn and Cu concentration that had the most significant impact on family richness. The graph on the left (A) shows the result of the CART analysis of dissolved Zn on value 14.23 μg/L as the initial split met the control factor (cp = 0.05), indicating the selected variable had a significant impact on family richness. The graph on the right (B) shows the result of the CART analysis, which found that 3.26 of μg/L dissolved Cu had the biggest influence on family richness. These figures split the data set into the boxplots that show the median along with the 25th and 75th percentile values.

Given the results from the GLMM-TMB-NS analysis, a classification and regression tree (CART) method was applied to identify the threshold value for the top environmental variables, Zn and Cu. Put simply, this method divides the data into two groups above or below a level, in this case, Zn, and then determines at which level the difference between the two groups has the greatest statistical significance. When a CART statistical model is used, and with the complexity parameter set at 0.05 (classification set at 0.005), it was found that a Zn level of 14.2 μg/L had the most significant effect on changing family richness (reducing levels below 14.2 μg/L could result in an increase of 8 families) (Figure 7A). Similarly, when the same CART approach and classification was repeated for Cu, it was found that a level of 3.3 μg/L had the most significant effect on changing family richness (Figure 7B). Note these levels identified in the CART analysis are telling us which concentrations were associated with the biggest impact on richness, not those which would be protective; this implies that neither 10 μg/L for Zn nor 4 μg/L for Cu would be sufficiently protective if chosen as Environmental Quality Standards (EQS).

There are many locations where low richness occurs even though Zn or Cu concentrations are low, and similarly, there are locations where high richness occurs despite concentrations of Zn or Cu exceeding toxic thresholds (Figure 7). Sites with poor richness, where the Zn or Cu are thought to be at nontoxic levels, will be where other factors are the driving pressure, such as perhaps BOD or ammonia, while locations with high, but apparently nontoxic effects from Zn and Cu, may be the result of reduced bioavailability of the metals associated with local water chemistry.

## Discussion

### Choices and Limitations in the Statistical Approach

This statistical analysis points to concentrations of Zn and Cu that have been, and still are, closely associated with overall macroinvertebrate richness in rivers in England at national scale. Nevertheless, it is notable that Zn and Cu did not have the same prominence for the EPT family richness (Figure 4) or in regions of high wastewater (Figure S1). We realize that all scientific studies have their limitations.^35^ With any study of this type, using historic field monitoring results, there can be issues with missing values, potential errors in reporting, misattribution of locations, assumptions on our part (that may be incorrect), on which statistic from the preceding 6 months data to use, and simple data inputting errors. Statistical associations fall short of causation, but the strength of association can form part of a weight of evidence approach.^36^

The analysis presented here has combined both temporal and spatial data. This choice was driven by the need to accurately account for the inherent spatiotemporal structure of the data. Disaggregating the components and including them as separate variables increases model complexity, leading to potential overfitting, especially in data sets where there are many missing values and few observations (Table S1). Overfitting may render some data sets unsuitable, reducing the number of viable models and the reliability of results. Additionally, disaggregating and retaining only one component may oversimplify relationships, fail to account for spatiotemporal structures, and miss important data patterns (see additional discussion on the topic in the Supporting Information). It would still be desirable for future research to aim at disaggregating the two components to provide a deeper understanding of the nature of the relationship between Zn and Cu and richness recovery, but this may require more data. However, as a first step, we addressed the temporal question by breaking down the statistical analysis into two time periods: pre-2006, when the greatest increase in overall family richness occurred, and post-2006, when little change in richness occurred. Zn and Cu remained as having the strongest associations for both periods (Figure 3). It is noticeable that in the second period, post-2006, the importance of BOD and ammoniacal-N fell with respect to both overall family richness and EPT family richness (Figure 3B). This probably reflects the national drop in BOD and ammonia concentrations following improvements in treatment by the water industry in response to the Urban Wastewater Treatment Directive. This might suggest the EPT group of macroinvertebrates, whose richness has continued to improve, is still benefiting from previous national reductions in gross biodegradable organic and nutrient loading.

Regarding the role of spatial (rather than temporal) factors dominating the results, we have started to consider this by eliminating the Zn-rich region of northern England from the data set, we have found this does not reduce the ranking of Zn for the remaining part of England. Many geographic variables are included in the statistical analysis, and the model was able to separate their importance from Zn/Cu.

### The Potential Roles of the Missing Variables of Organic Contaminants and Aluminum

A further limitation was that not every desirable chemical stressor could be included (such as specific organic contaminants or aluminum) in the statistical analysis. Long-term national monitoring of organic pollutants does not routinely measure individual organic pollutants in England. However, there are reasons to question whether organic pollutants, singly or together, even if routinely measured, could have played a more significant role than the grouping of Zn, Cu, BOD, or NH_3_. Noting the universal recovery in macroinvertebrate richness occurring across different landscapes since 1989 analyzed by Qu et al. (2023),^9^ to be critical national variables, the chemical(s) would have to be omnipresent in uplands and lowlands, and also present in low as well as high wastewater settings (rural and urban) in rivers (like the nutrients and metals). To fit the pattern of improving family richness, their concentrations should have been declining post-1990, before leveling off in the mid-2000 period, in rural as well as urban locations. The competing organic contaminants would also have to be less toxic to the EPT group of invertebrates compared to the other invertebrates (given the increasing EPT richness post-2000). The estimated wastewater contribution, which acts as a proxy for any organic pollutants discharged from domestic sources, is already included in these analyses, and yet, wastewater exposure was not ranked at the top. By including arable land as a land cover factor, this could be considered a proxy for pesticide exposure, as potentially might wastewater, yet neither the proportion of arable land in the catchment nor wastewater exposure was ranked as the most important variable. In England, the one region where a decline in family richness occurred post-2007, following an earlier slow improvement, was the north. In the north, unlike most other variables, Zn declined and then increased in concentration (see Figure S5). This does not mean that organic contaminants individually or as mixtures are not harming macroinvertebrates, but that their importance is restricted to local situations and therefore is less likely to be detected in a national analysis.

Aluminum (Al) is not routinely measured, and therefore, changes in its dissolved concentrations over time cannot be ruled out as having played a role. However, the toxicity of Al, one of the most common elements of the earth’s crust, is particularly related to acidity, and it has been considered to be more influential in acid headwaters than lower in the catchment.^37^ In this investigation, the mean river pH from 1989 to 2017 was 7.77, with the 25th and 75th percentiles being 7.52 and 8.05; in other words, the majority of English rivers over this period had a neutral or mildly alkaline pH.

### The Strength of the Case for Zinc and Copper and Their International Relevance

Although still only a relative analysis, this statistical examination points to levels of Zn and Cu as being more negatively associated than a wide range of other variables with overall macroinvertebrate richness at the national scale. It was found that Zn and Cu maintained their position at the top of the rankings for association with overall family richness, while the importance rankings of the other variables could alter, according to the type of analysis undertaken. This consistency suggests that the identification of these metals as important variables has some robustness. The analysis showed that where Zn levels fell below 14 μg/L, and Cu levels fell below 3.3 μg/L, the biggest changes in richness occurred. It must be re-emphasized that this analysis is giving the national and not the local picture. While we have dwelt on Zn and Cu as apparently very important for invertebrates, there remain others in close proximity to these metals. Thus, if it were possible to eliminate Zn and Cu from all waterbodies, the next steps would be to focus on the next set of variables that were identified as lower in importance, such as Ni, Fe, BOD, and the ammonia family.

How relevant might this analysis of an English data set be to what has happened further afield? A general increase in macroinvertebrate richness from more denuded states in the 1980s and early 1990s has been reported in North America^7^ and Continental Europe.^8^ That improvement in richness was followed by a slowing or plateau, which is particularly distinguishable in England^9^ and Continental Europe.^8^ The reduction in concentrations of metals like Zn or Cu from the 1980s to fairly stable levels from the late 1990s onward, as shown in this study, can also be observed in major European rivers.^38−40^ The similarity in the trends over time for both macroinvertebrate richness and metals in Continental Europe, as carried out here for England, is striking.

If Zn levels below 14 μg/L and Cu levels below 3.3 μg/L were associated with the biggest gains in English macroinvertebrate richness, how relevant are these concentrations for other countries? While European dissolved Cu concentrations in recent decades seem somewhat lower than those reported in England (around 1.0–1.5 μg/L),^38,41,42^ the dissolved Zn levels are similar or higher (around 5–11 μg/L).^41,43^ A nonexhaustive look across the world suggests that the English levels of Zn and Cu are not remarkable. For Asia (India, Japan, and China), levels in some major rivers can be higher, such as mean or median Zn levels of 9–30 μg/L and mean Cu levels of 1.3–4.7 μg/L.^44−48^ In the Americas, Zn levels of 25–120 μg/L are reported in Ecuador,^49^ 40–540 μg/L in Argentina, and 9–89 μg/L for Cu.^50^ Of course, we must recognize that for different countries, if other elements are at higher, more acutely toxic or more disruptive levels, such as for BOD, then Zn and Cu will be of secondary importance.^48^

### What Might Have Influenced Zinc and Copper Concentrations in Water?

It is reasonable to presume that the European Urban Wastewater Treatment Directive (UWWTD, Council Directive 91/271/EEC, implemented in 1991 with full compliance in 1998) played an important role in the reductions in gross organic pollution, ammonia, and nutrients in UK rivers.^1,12,13^ It should be acknowledged that nutrient levels also declined in rural areas, which may reflect more responsible and efficient farming practices.^9^ The changes or declines in the concentration of metals cannot be solely attributed to the UWWTD, and may reflect reductions in atmospheric pollution associated with the end of coal-burning,^51^ with the concomitant increase in soil pH,^52^ a decline in heavy industry,^13^ and possibly also some reduction in society’s domestic consumption of metal products.^13^

It is still necessary to identify what features of urban land cover are or were so detrimental to macroinvertebrate diversity (although that suppressing effect is lessening slightly with time).^9^ As shown in the statistical analyses, the negative influence of urban land cover can be distinguished from wastewater (and Zn) or habitat modification score. Interestingly, transient and episodic runoff from urban areas can have very high Zn (and Cu) levels, up to 100s of μg/L,^40,53^ which would be recognized as highly toxic,^54^ but these events would be unlikely to be detected by routine river sampling.

### Integrated Monitoring Programs Combined with Statistical Analyses Might Ensure Better Outcomes for Wildlife

The way priority chemicals are currently identified for action, ensuring aquatic wildlife may be better protected, could be described as “top-down”. That approach uses laboratory ecotoxicity data (typically short-term laboratory tests on a relatively small number of species) and river measurements or predictions to generate a list of chemicals of concern.^55,56^ However, there is little field confirmation that this approach is either under- or overprotective.^57^ Here, we used a “bottom-up” approach, relying on a statistical analysis of large wildlife and stressor field data sets (consistent monitoring by regulatory agencies being critical to this approach) to identify factors that are most closely associated with biodiversity. We suggest that this approach has considerable merit and at the very least can act as a sense check on the traditional approach.

This statistical analysis, which was uninhibited as far as possible by any *a priori* assumptions, revealed Zn and Cu as potentially among the most important stressors of river invertebrates over the past 30 years, and deserving much greater attention. Previously, a totally different methodology came to a similar conclusion as to their high relative risk for English rivers compared to other chemicals.^34^ The full integrated data set which the project pulled together in preparation for the statistical analysis is now publicly available to support further research.^20^

## Supporting Information Available

Information about the data set, additional statistical methodological details, uncertainty, and rationale of the methods; number of the tested environmental variables’ observations and the percentage of the missing values; overall changes in family richness; ranking variable importance by different wastewater levels; results from the GAMM model; chemical concentration trends in northern England, and the geographic distribution of Zn and Cu; ranking variable importance excluding northern sites (PDF)
