# Supplementary material for: Zinc and Copper Have the Greatest Relative Importance for River Macroinvertebrate Richness at a National Scale
Source: Environ Sci Technol. 2025 Feb 17;59(8):4068–79. doi: 10.1021/acs.est.4c06849 (PMC11883809; doi:10.1021/acs.est.4c06849)
Supplement: Supplementary file 1 — es4c06849_si_001.pdf [file es4c06849_si_001.pdf]

Supporting Information

Zinc and copper have the greatest relative importance for river macroinvertebrate richness at a national scale

Andrew C. Johnson[1, *] Dinara Sadykova[1], Yueming Qu[1], Virginie D.J. Keller[1], Nuria Bachiller-Jareno[1,2], Monika D. Jürgens[1,] Michael Eastman[1,3], François Edwards[1,4], Clarissa Rizzo[1,6], Peter M. Scarlett[1], John P. Sumpter[5]

[1] UK Centre for Ecology and Hydrology, Wallingford, OX10 8BB, UK

[2] University of Exeter, Exeter, EX4 4QJ, UK

[3] Met Office, Exeter, EX1 3PB, UK

[4] APEM Ltd, Chester, CH4 0GZ, UK

[5] Brunel University London, UB8 3PH

[6] Wallingford Hydrosolutions, Howbery Business Park, Wallingford, OX10 8BA

[*]**Corresponding author**: ajo@ceh.ac.uk

Number of pages 26

Number of figures: 6

Number of tables: 1

Introduction to the data

The data used in this study is available in the NERC Environmental Information Data Centre (EIDC) as *Macroinvertebrate taxonomic abundance, water quality, river flow, air temperature and environmental site descriptors from English rivers, 1965 – 2018* data product [1]. More complete descriptions of the methodology to assemble this product and file format can be found in this data product from which the descriptions provided below have been summarised. The basis of the assessment was the Environment Agency's (EA) Freshwater macroinvertebrate surveys datasets (BIOSYS) publicly available from the Ecology and Fish data explorer (https://environment.data.gov.uk/ecology/explorer). This dataset contains metric and taxa information from freshwater river macroinvertebrate surveys carried out across England from the mid-1960s onwards. As well as the macroinvertebrate data this dataset also includes latitude, altitude, slope, river discharge category, and distance from source.

The EA standardized the method of macroinvertebrate surveys and introduced quality assurance procedures in 1990 (John Murray-Bligh, pers. comm.). Some of the sites surveyed may have been visited only a few times to record macroinvertebrates, whilst others have a consistent set of records going back over 30 years, with up to four samples per year. Of the sites with the longest records, a sub-set of 1,457 from across England were selected which could be matched to nearby chemical and physical (flow and temperature) monitoring sites to permit the statistical analysis. These selected sites were considered representative of all regions and their macroinvertebrate trend results mirror those found at the national scale with the full dataset [2].

Aquatic macroinvertebrate biomonitoring data

To describe the biodiversity of macroinvertebrates across these sites over time, we considered only richness at the family level. The analysis focused on presence-absence recording at the family level, which was consistent over the recording period. For this statistical analysis we focused on:

- Family richness: this is the number of families present at a site. One family may include several species. It is an indicator of the overall taxonomic community status. Oligochaeta were not differentiated further, despite different families having different tolerances to environmental quality.
- Ephemeroptera (mayflies), Plecoptera (stoneflies), Trichoptera (caddisflies) family richness (EPT_FR): this is the total number of families within three major orders of stream insects that are considered to be relatively sensitive to water pollution. This index is a variant of the EPT ratio, which is often used internationally as a useful measure of water quality [3,4].

River Flow Gauging Station Matching and flow statistics

To derive relevant flow statistics, the most appropriate gauging station had to be assigned to each macroinvertebrate site. This was the gauging station that is nearest to the macroinvertebrate survey site according to distance along the river network which was calculated in Python 3. The process required the gauging stations to be added to the UKCEH 1:50 000 Scale Digital River Network [5]. Gauging stations were snapped to the nearest line on the river network according to Euclidean distance. The equivalent process is then completed for the macroinvertebrate sites on the river network that contains the gauging stations separately. The distance along the network between each macroinvertebrate survey site and connected gauging station is then calculated, and the nearest assigned to the survey site. The Euclidean distance for the results of this exercise showed the maximum distance between macroinvertebrate site and flow gauging site was 124.9 km, the mean was 6.7 km, the median was 4.7 km.

The mean, median, sample standard deviation, coefficient of variation, Q5/mean and Q95/mean were calculated over the full period of interest (from the earliest macroinvertebrate sample date minus six months preceding period to the most recent sample date used: 1974/05/20 – 2019/03/29). Also, several threshold statistics were calculated for the 6-month period preceding each macroinvertebrate survey.

In our full statistical analysis, we only used the 5% percentile threshold flow variable, representing low flows (equivalent to 95% percentile in hydrology). The other seven flow variables showed a similar ranking in the variable importance and are therefore not presented in the main results. This hydrological information could be linked both spatially and temporally to macroinvertebrate sites and sample dates.

Estimating land-cover upstream of macroinvertebrate sites

The land-cover values upstream for each macroinvertebrate sample point were extracted using an National River Flow Archive bespoke Python script and the Integrated Hydrological Digital Terrain Model (IHDTM) [6]. The IHDTM is used to identify the upstream area draining through a macroinvertebrate monitoring point. The UK Centre for Ecology and Hydrology Land Cover Map for 2015 was used. From the IHDTM output, the 21 land cover types mapped in the Land Cover Map 2015 (25 m raster) [7] were grouped into four large categories: woodlands, arable/horticulture (termed arable although it includes horticulture), semi-natural and urban land cover types and their percentage covers in the upstream catchment were calculated. This information was linked spatially to macroinvertebrate sites.

Predicting the wastewater exposure at each macroinvertebrate site

As an indication of exposure to the range of contaminants discharged from municipal wastewater, we used a GIS-based water quality model, LF2000-WQX (LowFlows2000 Water Quality eXtension) model [8]. This spatially explicit model predicts flow throughout catchments based on a 40-year climate record and combines this with location and flow (consented dry weather flow) of Wastewater Treatment Works (WwTWs) provided by the UK Water Industry in 2008 when the model was established. When tested against a known human contaminant, such as steroid estrogens, its predictions for concentrations within rivers have been well within an order of magnitude of the field measurements [9-11]. In this case the given WwTW dry weather flow of each plant was used to represent the treated wastewater effluent. The predicted wastewater percentage only considers the contribution of those WwTWs that contribute 90% of the dry weather flow discharged in a catchment (very small WwTWs and septic tanks are, therefore, not considered). Each macroinvertebrate site was assigned the wastewater value predicted for that exact river reach.

Collecting chemical data from nearby water quality sites (EA Water Quality Archive)

To derive relevant water quality statistics, the most appropriate Environment Agency water quality Archive site was assigned to each invertebrate monitoring site. A set of chemical concentration data from the period of 1/1960-11/2015 was provided to UKCEH by the EA as individual files which were stored in-house on an Oracle database implemented for that purpose. The Environment Agency has made most of their water quality monitoring data for England from 2000 onwards freely available at https://environment.data.gov.uk/water-quality/view/download. Thus, in June 2020, all the then available data was downloaded as annual files for "all of England" and "both compliance and monitoring" which was used to replace the generally less complete post 2000 data in the originally supplied files. After plotting the locations of both the selected macroinvertebrate monitoring sites and the water quality monitoring sites as well as the river network in ArcGIS, the appropriate water quality sites were identified. The UKCEH IRN Blueline extension for ArcGIS [12] was used to help find the nearest matches along the river network. The outcome was checked manually to choose between candidate sites upstream or downstream of the invertebrate site. The matched water quality sites were finally chosen according to the following guidelines:

1. The site matching was performed on the originally supplied dataset up to 2015
2. Only water quality sampling sites corresponding to a freshwater sampling point type (SMPT_TYPE starts with F)
3. Only sites where the original dataset up to 2015 had at least 25 samples of the material code 2AZZ (river/running surface water) were considered.
4. Sampling purpose COMPLIANCE and MONITORING were both included.

5. The water quality site must be on the same river or stream as the wildlife monitoring site but can be upstream or downstream.
6. No marked wastewater treatment works exist between the quality sampling site and the invertebrate monitoring site (very small works could have been missed – see above).
7. No major tributaries exist between the quality sampling site and invertebrate site.
8. Where there was a choice between two or more acceptable sites, a decision was made, based on minimizing the size and number of tributaries and distance while maximising the number of measurements.

On average, it was found the selected chemical monitoring site was 76 m from the macroinvertebrate sampling site. Once the sites had been matched, the data for the selected chemicals was downloaded. Simple statistics were calculated at each water quality site as minimum, maximum, mean, and standard deviation. These statistics were calculated for an antecedent period of 6 months prior to the macroinvertebrate survey date.

For entries described as below the limit of quantification (LOQ) we reviewed the influence of deciding whether the <LOQ=LOQ value (i.e. "worst case"), <LOQ=0 (i.e. "best case") or <LOQ=LOQ/2 (most commonly used approach) was most appropriate. We tested these alternatives across a substantial number of models. The results were very similar, and so we stuck with <LOQ=LOQ/2 in the paper.

The % of values below LOQ as well as the range of LOQs reported over time is given in Supporting Table 1 (The dataset only reports LOQ, when the measurement is below it, e.g. an entry of "<0.1 μg/L" indicates that the LOQ was 0.1 μg/L on that occasion, but it is not possible to know for certain where the LOQ was for entries above it). There was a general trend for the LOQ's to improve, i.e. get smaller, over time. One danger with substituting <LOQ in this situation with LOQ/2 for the statistics is, that the generally reducing LOQs would then produce an artificially decreasing trend where there is a large proportion of values <LOQ. However, the alternative approach of setting the <LOQ values to zero was also tested and yielded very similar results. In that scenario one would imagine artificially increasing values as over time fewer zeros are reported with improved quantification limits.

Here we would like to add that Zn(d) with 28% and Cu(t) with 17% values <LOQ seem less affected by changing LOQs over time, supporting the robustness of conclusions drawn for these metals as their concentrations are less likely to be skewed. Conversely, metals such as Hg, Cr, and Cd have the highest percentages of values below the limit of detection (%<LOQ) at 60% or more, with Pb and Ni close behind in the high 50s%. For these metals, results may be more sensitive to changes in LOQs over time.

Note, this chemical information could be linked both spatially and temporally to macroinvertebrate sites and sample dates.

Air temperature

It would have been desirable to include water temperature in the statistical calculations, but this has not been consistently recorded, so air temperature was used as a proxy. Air temperature data to accompany the macroinvertebrate observations were sourced from the Climate Hydrology and Ecology Research Support System meteorology dataset (CHESS-met), a gridded dataset with a 1 km resolution, spanning from 1961 to 2017 [13]. For each macroinvertebrate survey location daily air temperature time series, corresponding to the CHESS-met grid cells enclosing the survey locations, were extracted. These values were then converted from Kelvin (K) to degrees Celsius (°C). The simple statistical metrics, of mean, median, minimum, maximum, and standard deviation for the 6 months leading up to the macroinvertebrate sampling date were calculated for the extracted time series. This temperature information could be linked both spatially and temporally to macroinvertebrate sites and sample dates.

Habitat quality and channel modification

Habitat quality and channel modification indices to accompany the macroinvertebrate data were sourced from the EA River Habitat Survey (RHS) database [14,15]. These surveys were conducted between 1994 to 2017 using standard Environmental Agency methodology. The habitat modification score (HMS) is derived partially from the extent of bank and bed reinforcement and modification, thus, the Bank and Bed Resectioning is a sub-score of HMS (HMS_RBBSS). The mean substratum (Msubst) quantifying riverbed status is a variable in RIVPACS (River InVertebrate Prediction And Classification System) programme. The value is calculated from the proportion of four bed substrates (boulders, pebbles, sand, and silt).

$$Msubst = -7.75 \times Boulder - 3.25 \times Pebbles + 2 \times Sand + 8 \times Silt$$

The RHS surveys are carried out along a 500 m length of watercourse by gathering observations on channel features and modifications at 10 equally spaced spot-checks, together with an overall summary for the whole site [16]. A copy of this dataset was downloaded from the www.gov.uk portal and processed to extract the river habitat quality data. To assign a modification score to a macroinvertebrate monitoring site, GIS-based extraction procedure was used to spatially match them to the macroinvertebrate sites[12].

Final selection of parameters for statistical analysis

Twenty-six of the shortlisted water quality parameters had enough data available for the chosen sampling sites to take forward for statistical analysis (Table 1). Those excluded organics such as PAH, Pesticides, DDT, were because over or equal to 91% were missing from the accompanying macroinvertebrate observation. This selected cut-off value was primarily motivated by the issue of model overfitting, as the integration of these variables with others resulted in very small datasets. Overfitting refers to a situation where a statistical model is capturing noise rather than genuine patterns. This can result in an inflated goodness-of-fit measure. While the overfitted model may

appear to explain a large portion of the variability in the observed data, this explanation is driven more by noise than by true relationships in the underlying population.

A high number of missing observations (with over or equal to 91%) might still be sufficient for a **simple** model but considering the complexity of the approach presented in the paper – including non-linear effects of all the variables, random effects, a temporal structure, and seasonality (the full model description is provided below) – it was insufficient for this modelling approach. The threshold of 91% was calculated using cross-validation techniques. Cross-validation is a commonly used technique to check for overfitting in statistical modelling, which assesses how well the model generalizes to new, unseen data. The threshold of 91% was determined to be the one that yielded models without overfitting.

Selecting suitable toxicity thresholds for Zn and Cu in rivers

Zn and Cu have been the subject of considerable research over the years regarding the levels that cause toxic effects in freshwater organisms. It is recognised that their toxicity can be reduced by complexation with organic matter, precipitation with $CaCO_3$, or the proportion present in the ionised form related to pH [17]. However, the actual degree to which these factors reduce toxicity in reality may not always align with the predictions [18]. For Zn an HC5 of 10 µg/L is calculated by Mebane, et al. [19], others suggest 11 µg/L would be a suitable environmental quality standard (EQS) [20,21]. The lowest EQS (set by $CaCO_3$ concentration) in Europe varies, but commonly start at 8 µg/L. Here we chose a minimum toxicity threshold level of 10 µg/L Zn against which to display English Zn values. In conditions of 1 mg/L DOC, 150 mg/L $CaCO_3$ and pH 7.5, an EC20 for Cu of 4 µg/L has been suggested [18], a value similar to that offered as an HC5 of 2 µg/L [19] whilst earlier publications have a NOEC at 4 µg/L [22]. Therefore, we selected 4 µg/L Cu as our minimum ecotoxicity threshold.

Do the results change if a different statistical approach is used?

Although this study focused on the use of a GLMM-TMB-NS statistical model, there are other competing approaches used in ecological analyses, such as Generalised Additive Mixed Models (GAMM) [23,24]. Thus, to examine whether a different methodological approach would offer the same ranking of variables, we used GAMM (gamm4 function in R). However, as the model had difficulty running with five separate variables because of convergence issues, in this case we used two chemical variables and one other variable (HMS/physical/land) (in addition to yearly trend effect, random effects and seasonal effect). GAMM also identified Zn and Cu as the leading variables, closely followed by ammonia and BOD (Supporting Fig.).

We also tested spatio-temporal models with integrated nested Laplace approximation (INLA) combined with Stochastic Partial Differential Equations approach (INLA-SPDE), using five variables. INLA-SPDE explicitly takes spatial autocorrelation into account, and we used geographic boundaries to ensure that spatial autocorrelation was accurately represented. However, because of their

computational intensity (the computational cost of models with INLA is exponential with respect to the number of parameters of prior distributions), we were only able to run a very limited set of models. Nonetheless, the results for these selected models showed very similar ranking to the same models with GLMM-TMB-NS, providing robustness to our importance variable results.

Additionally, investigating GLMMs-TMB-NS and GAMMs, a comprehensive comparative analysis was undertaken to assess the influence of modifications applied to a core model, such as the number of chemical variables considered (from 2 to 6), the number of physical/land and other variables included within a single model (from 0 to 3), whether random effects are nested or not, etc. Regardless of the specific modifications considered, notably similar results in terms of variable importance were obtained. While variables with closely matched values could interchange positions in the importance table, the leading variables consistently retained their status as the foremost contributors. This observation underscores the robustness of the findings, revealing that the nuances within the main model did not significantly impact the overall results.

Uncertainty

A limitation of the study is uncertainty, which can arise from multiple sources, encompassing the data collection process with potential errors or biases, modifications introduced during pre-processing, and various factors related to modelling choices. Additionally, external influences and inherent variability under study contribute to the overall uncertainty. Acknowledging these diverse sources of uncertainty is essential for a comprehensive understanding of the results.

Assessment of autocorrelation and model assumptions

To assess spatial autocorrelation, we calculated Moran's I statistic for a representative subset of datasets, focusing on the measure of spatial dependency in Family Richness. The analysis revealed consistently positive Moran's I values across all evaluated datasets, with a minimum statistic of 11.50 and a maximum of 37.51, indicating significant spatial clustering of similar values. The p-values for all tests were extremely low ($p < 2.2e-16$), confirming the presence of significant spatial autocorrelation. These findings suggest that sites with similar macroinvertebrate richness tend to cluster geographically, which may influence the results of any subsequent analyses. Given the substantial evidence of spatial autocorrelation, it was crucial to incorporate spatial effects into the modeling framework to ensure robust and unbiased estimates of the relationships between environmental variables and macroinvertebrate richness.

While spatial nested random effects (sites within regions) do not explicitly account for spatial autocorrelation, our analysis suggests that these models efficiently captured spatial patterns in our case. Moran's I tests on model residuals (after modelling was applied to datasets) consistently yielded non-significant p-values ($p > 0.1$), often around 0.9-1.0, and Moran's I statistics close to zero or negative. This indicates that our models have accurately incorporated the spatial dynamics, with p-values

indicating no significant remaining spatial structure. To further confirm this, we tested a subset of models using INLA spatio-temporal models, which explicitly account for spatial autocorrelation. The results from these models were highly similar to our original models, providing additional evidence that spatial autocorrelation was not a major concern in our analysis.

For temporal autocorrelation assessment, we examined residual autocorrelation using ACF and PACF plots in conjunction with Ljung-Box tests for a representative subset of model. Results from ACF and PACF plots indicated that temporal autocorrelation was largely mitigated; however, minor residual autocorrelation could remain at lower lags for some models. To confirm that this residual autocorrelation did not adversely affect model performance, we conducted cross-validation with RMSE and MAE metrics, comparing our full models against intercept-only models and models without temporal structures and random effects. The full models consistently demonstrated superior performance relative to these simpler models, suggesting that the remaining low-lag autocorrelation did not substantively impact predictive accuracy.

Additionally, to further ensure that minor residual temporal autocorrelation of our GLMMs-TMB-NS did not compromise model performance, we compared our models with a subset of more complex generalized additive mixed models (GAMMs) that incorporated ARMA structures, which showed no remaining residual autocorrelation (using this structure in modelling: correlation = corARMA(form = ~1|Year, p=X, q=Y) from the nlme library in R, incorporated within a gamm() model from the mgcv package), with values of p and q ranging from 1 to 3. Due to their complexity, however, these GAMM models faced severe convergence issues, preventing their use across all models and limiting this analysis to a small subset where convergence was achievable. Nevertheless, for the subset where these GAMM models converged, they produced nearly identical variable importance results to our models, and cross-validation results showed predictive accuracy on par with, or sometimes slightly better/worse than our GLMM-TMB-NS models - depending on the model applied. This comparison reinforces that our GLMM-TMB-NS models are robust in predictive ability and that the small residual autocorrelation is not of concern.

To ensure that the model assumptions were adequately satisfied, we evaluated various aspects of model fit and structure on a representative subset of models. Our negative binomial models were built on the assumption of an appropriate log-link specification, which we assessed through the analysis of residual plots: the absence of systematic patterns indicated that the functional form was suitable for our data. To address the issue of overdispersion, we conducted a comparative analysis of Poisson and negative binomial models (with linear parametrization), finding that the latter significantly enhanced both the Akaike Information Criterion (AIC) values and overall model diagnostics. Furthermore, we implemented cross-validation as an additional measure to evaluate model fit, performance, and potential overfitting. The results of the cross-validation demonstrated that the models generalized well to unseen data with no sign of overfitting, thereby reinforcing the robustness of our analytical approach.

Rationale

Imputation methods

In this subsection, we explain our decision not to employ imputation methods to address missing values, despite the common recourse to imputation techniques in similar studies. Our dataset revealed a notably high prevalence of missing values, particularly among the 15 metals examined. Specifically, 13 of these metals exhibited rates exceeding 70% of missing values, while only Cu dissolved, and Zn total had 57% and 52% missing values respectively (Supporting Table 1). All the missing values accounted for a substantial portion of the dataset, encompassing a total of 41 variables under consideration. This significant proportion of missing data presented a considerable challenge, necessitating a careful evaluation of potential imputation strategies.

We explored various strategies to ensure robust and unbiased analyses. This exploration included investigating imputation techniques such as Bayesian principal component analysis (BPCA) with data imputation, alongside traditional imputation methods. However, our thorough validation process, which encompassed cross-validation and simulation studies, revealed significant challenges associated with imputation in our specific context. Despite efforts to select the best models, those incorporating imputed values consistently exhibited poor predictive performance. Furthermore, simulation studies underscored the potential for imputation to introduce bias in variable importance estimates, particularly in the face of high rates of missing data.

Additionally, previous studies have indicated that while imputation techniques may demonstrate success for certain chemicals commonly found in geological formations, such as calcium carbonate ($CaCO_3$), their applicability to elements like Zn or Cu would have some risk because of unpredictability regarding their sources, thanks to historic point sources from old mines. Therefore, caution is warranted when considering imputation for chemical variables.

Therefore, we opted for a systematic approach of considering all possible combinations of variables, ensuring that no data observation was overlooked and minimizing the risk of biased results.

A common question arises regarding our decision to include all 41 variables, despite their high prevalence of missing values. This choice was guided by several key considerations. Primarily, our study aimed to investigate the complex interplay between a wide range of chemical and non-chemical variables and their collective impact on the Family Richness (or EPT Richness). By considering all available variables, we aimed to capture the full spectrum of potential influences. Furthermore, omitting variables based solely on their missing values prevalence could risk overlooking important contributors and limit the scope of our analysis. Additionally, we tried to minimize the risk of selection bias and ensure that our analysis captured the full complexity of the underlying data structure.

Integration of Spatial and Temporal Dynamics

It is essential to acknowledge the existence of various approaches within ecological studies. One of the recent approaches in analogous studies advocates for disentangling spatial and temporal effects (e.g., Oedekoven, et al. [25]). However, our approach in this study, which focuses on understanding the individual contributions of variables to Family Richness (or EPT Richness), diverges from this recommendation without explicitly dividing the explanatory variables into spatial and temporal components.

While disentangling spatial and temporal effects can be valuable in certain contexts, we opted for a comprehensive approach that considers the overall spatial and temporal patterns in the data. We found out that dividing the data into spatial and temporal components adds complexity in our already complex study with 41 variables and many missing values without necessarily providing additional insights relevant to our specific research objective. Given the potential challenges associated with increased model complexity, such as overfitting and computational burden, and the limited size of some of our datasets, again because of missing values, we determined that the benefits of this approach may not outweigh the potential challenges it presents.

By incorporating spatial random effects, accounting for seasonality, using a temporal covariance structure, including land variables, etc., our approach allowed the capture of spatial and temporal dynamics in the data while focusing on the primary objective of identifying the most important factors. The inclusion of non-linear effects in our modelling framework further enhances our ability to capture the complex relationships between the considered variables and Family Richness (or EPT Richness). This comprehensive framework provides valuable insights into the ecological processes at play, even if it does not explicitly separate spatial and temporal effects into distinct axes at the level of explanatory variables. We did test this disaggregation approach on a very small number of models with a large number of observations and found that it provided similar results, indicating that the combined approach captures the necessary relationships effectively and results in consistent assessments of variable importance.

Supporting Table

Supporting Table 1. The Variables, number of macroinvertebrate observations with corresponding (6 month average) observations of this variable and the percentage of missing values, sorted by missing value percentage. The percentages are given in relation to the total number of invertebrate observations, 65,032. Note: individual percentages lack specificity about missing values in any particular model, as any set of five variables within each model may exhibit missing values at different locations or points in time. There is no statistically significant correlation (correlation coefficient of 0.1) between the percentages of missing values and the variable importance values provided in Figure 2. The LOQ for each chemical determinand (which generally improved over time) and the percentage of measurements outside the method range is also given. These are normally where concentrations were too low to be quantified (<LOQ) but include for some parameters (e.g. BOD) a small number of measurements where the value was too high *for the measurement method. Note, that this percentage is for the whole number of measurements used for the 6 month averages and includes some duplicate entries, where two invertebrate samples were taken less than 6 months apart: For example, if invertebrates were monitored in May and September and some other parameters were measured at the beginning of each month, then the May sample would be associated with the 6 months previous i.e. December to May monitoring and the September sample with April-September, making it 6 entries for each and thus 12 in total, where the April and May measurements are used twice and thus also counted twice, when the percentage outside the measurement range is calculated.*

Variable	Number of observations	Percentage of missing values	LOQ	% < LOQ (or occasionally > upper measuring limit)
Hg	6,441	90%	0.002 - 0.5 µg/l	63%
Cd(d)	7,284	89%	0.01 - 100 µg/l	69%
Fe(t)	8,614	87%	≤ 1 - 1000 µg/l	1.91%
Fe(d)	8,418	87%	≤ 1 - 290 µg/l	20%
Cr(d)	8,972	86%	0.001 - 60 µg/l	64%
Pb(d)	9,119	86%	≤ 0.003 - 70 µg/l	56%
Ni(d)	9,200	86%	0.005 - 50 µg/l	53%
Zn(d)	9,005	86%	0.05 - 50 µg/l	28%
Cr(t)	12,013	82%	0.001 - 50 µg/l	48%
Ni(t)	11,789	82%	0.005 - 50 µg/l	47%
Pb(t)	12,071	81%	≤ 0.002 - 100 µg/l	35%
Cd(t)	13,016	80%	0.001 - 100 µg/l	60%
Cu(t)	14,964	77%	0.01 - 50 µg/l	17%
Hardness	19,500	70%	1 - 40 mg/l	0.02%
Cu(d)	28,078	57%	0.01 - 30 µg/l	24%
Zn(t)	31,492	52%	0.01 - 50 µg/l	24%
SuspendedSolids	35,046	46%	0.1 - 10 mg/l	17%
NO3	36,525	44%	0.004 - 14.5 mg/l (as	2.59%
Dissolved O2	38,060	41%	≤ 0.286 - 10 mg/l	0.02%
Alkalinity	38,708	40%	1 - 50 mg/l	3.27%
NO2	40,499	38%	0.001 - 0.1 mg/l (as	15%
O2 saturation	40,481	38%	≤ 0.4%	0.03%
BOD	44,099	32%	0.01 - 28.5 mg/l	17%
NH3	47,086	28%	0.00001 - 0.02 mg/l	35%
PO4	47,017	28%	0.0001 - 2 mg/l	16%
Flow	49,562	24%	N/A	N/A

Ammoniacal-N	52,903	19%	0.001 - 1 mg/l (as N)	35%
pH	52,759	19%	N/A	0
HMS	56,185	14%	N/A	N/A
HMS RBBSS	56,185	14%	N/A	N/A
Bed substrate	63,510	2%	N/A	N/A
Temperature	64,077	1%	N/A	0
Altitude	64,907	0	N/A	N/A
Arable	64,834	0	N/A	N/A
Flow discharge	64,907	0	N/A	N/A
Dist.from source	64,937	0	N/A	N/A
Seminatural	64,834	0	N/A	N/A
Slope	64,849	0	N/A	N/A
Urban	64,834	0	N/A	N/A
Wastewater	65,032	0	N/A	N/A
Woodland	64,834	0	N/A	N/A

Supporting Figures

Variable Importance by Wastewater Levels

Family Richness

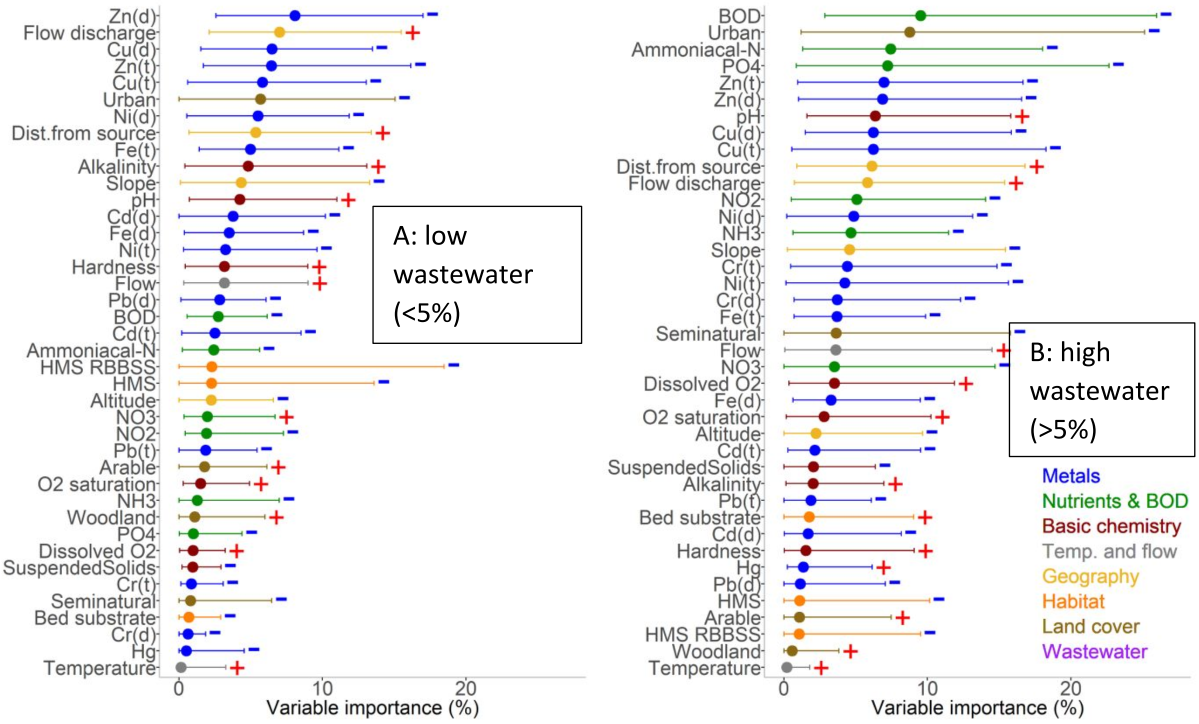

Supporting Fig. 1. Relative importance of variables from the GLMM-TMB-NS statistical model for macroinvertebrate family richness in England; based on 1,457 sites. The figure presents the percentage of explained deviance by each variable for sites with wastewater less than 5 percent (A; left) (46,871 observations) and for sites with wastewater greater than 5 percent (B; right) (18,161 observations). Dots represent mean values across models. Lines indicate the range of explained deviance, with the left and right parts corresponding to minimum and maximum percentages for each variable, respectively. Positive or negative relationships are denoted by plus and minus signs.

EPT Family Richness

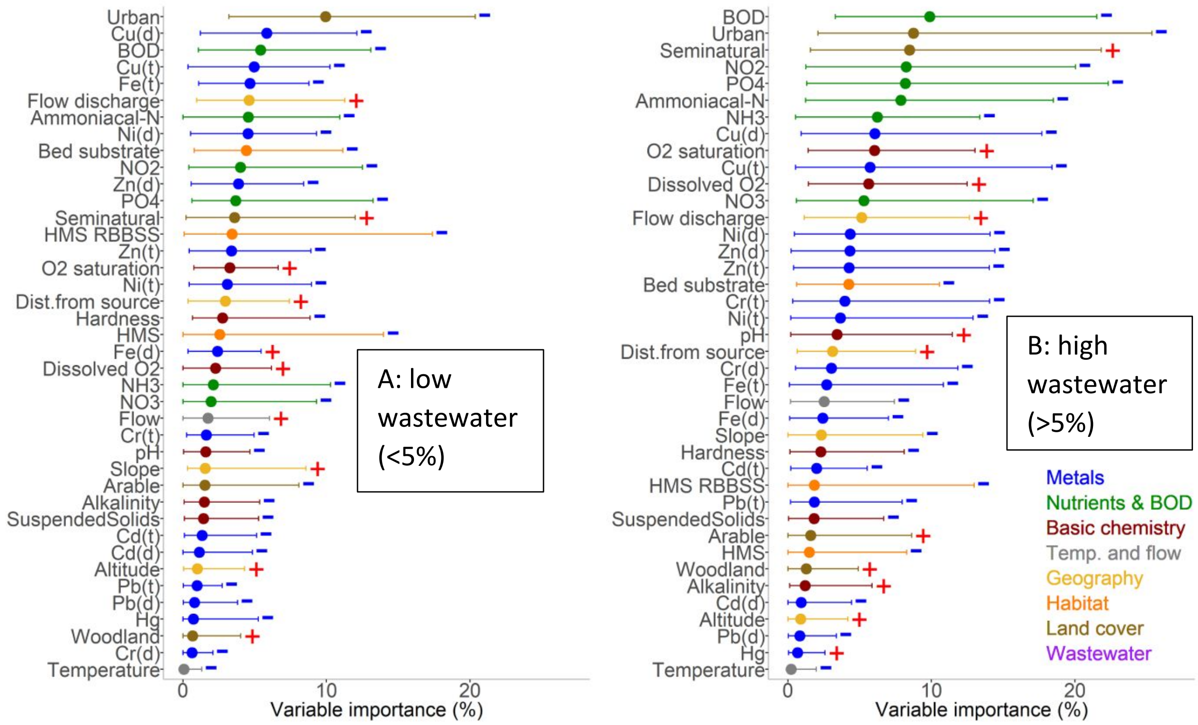

Supporting Fig. 2. Relative importance of variables from the GLMM-TMB-NS statistical model for macroinvertebrate EPT family richness in England; based on 1,457 sites. The figure presents the percentage of explained deviance by each variable for sites with average predicted wastewater concentration less than 5 percent (A; left) (46,871 observations) and for sites with wastewater greater than 5 percent (B; right) (18,161 observations). Dots represent mean values across models. Lines indicate the range of explained deviance, with the left and right parts corresponding to minimum and maximum percentages for each variable, respectively. Positive or negative relationships are denoted by plus and minus signs.

GAMM statistical model

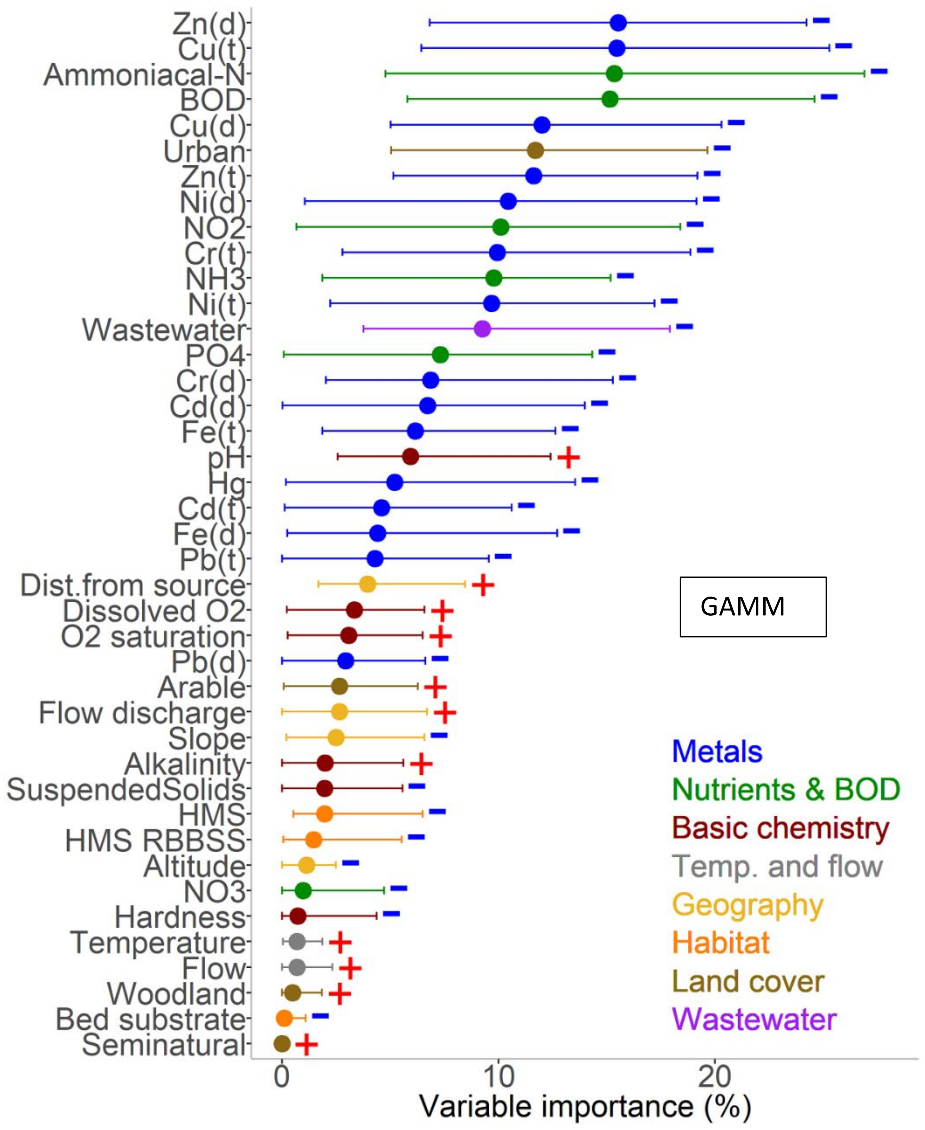

Supporting Fig. 3. Relative importance of variables from the GAMM statistical model for macroinvertebrate family richness in England; based on 1,457 sites (65,032 observations). The figure presents the percentage of explained deviance by each variable. Dots represent mean values across models. Lines indicate the range of explained deviance, with the left and right parts corresponding to minimum and maximum percentages for each variable, respectively. Positive or negative relationships are denoted by plus and minus signs

Chemical concentration trends in northern England

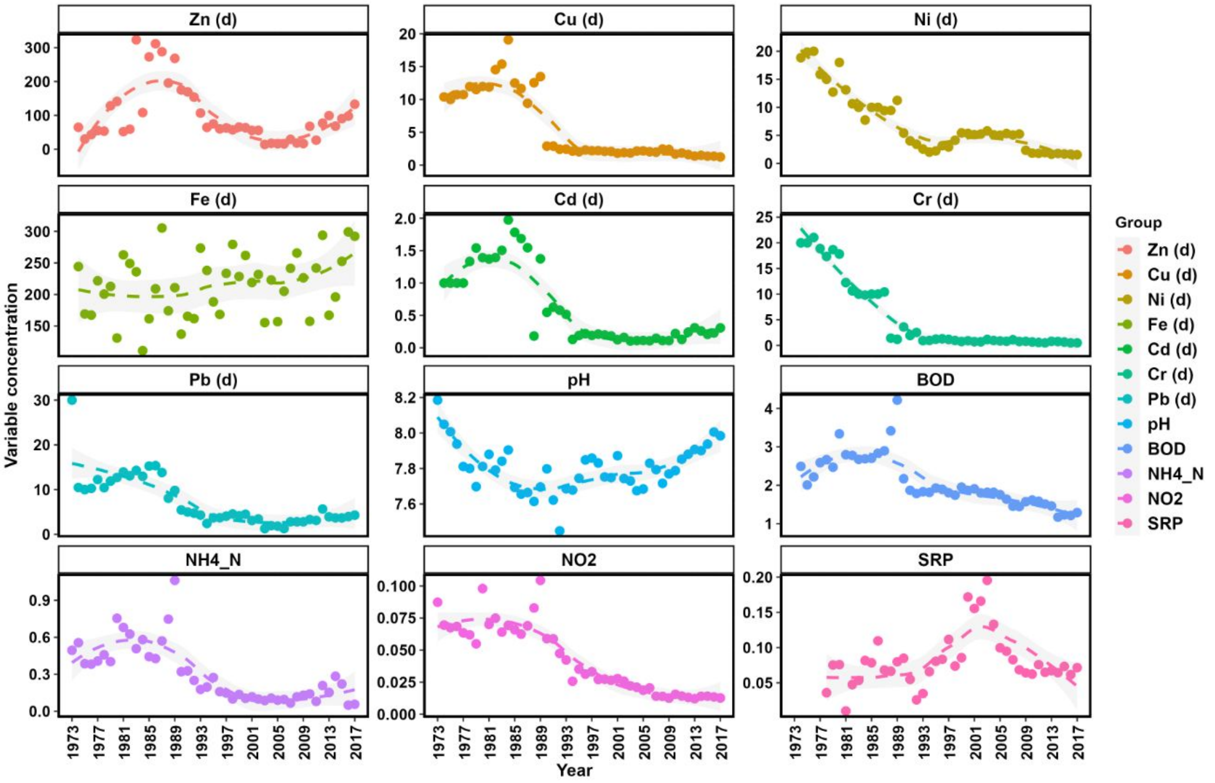

Supporting Fig. 4. Trends in mean annual concentrations of dissolved Zinc, Copper, Nickel, Iron, Cadmium, Chromium, Lead, pH, BOD, Ammoniacal-Nitrogen, Nitrite, Orthophosphate at the macroinvertebrate sites over the period 1973-2017 for northern England (latitude above 54.5°).

Geographic distribution of Zn and Cu

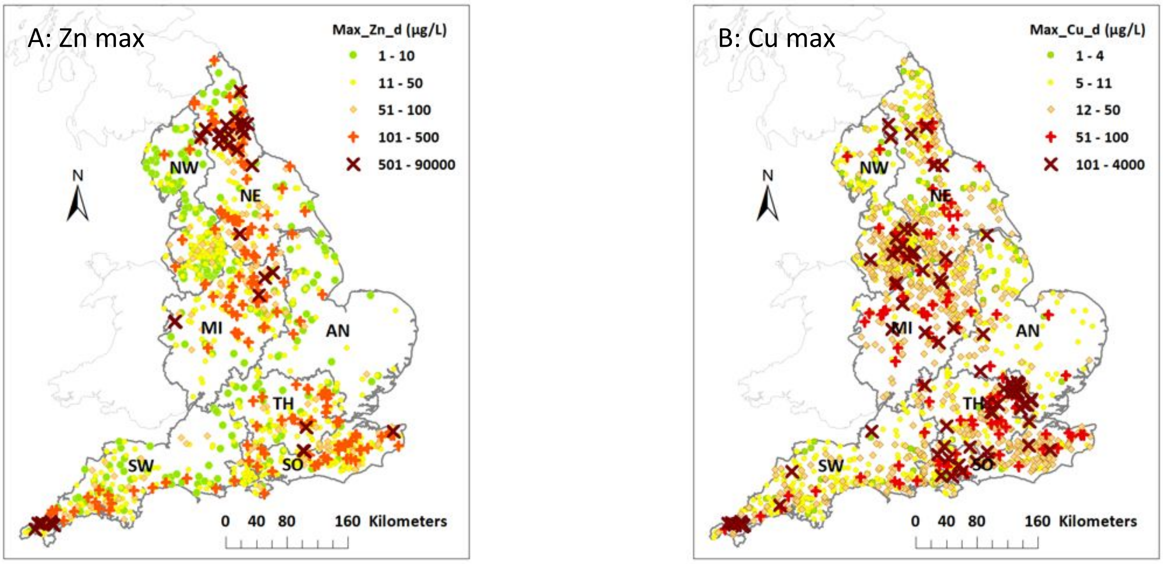

Supporting Fig. 5. Distribution of the maximum Zn or Cu levels experienced at any of the macroinvertebrate sites over the period 1974-2017.

GLMM-TMB-NS without northern sites

(Most of the Zn hotspots are in the north, see Supplementary Figure 6)

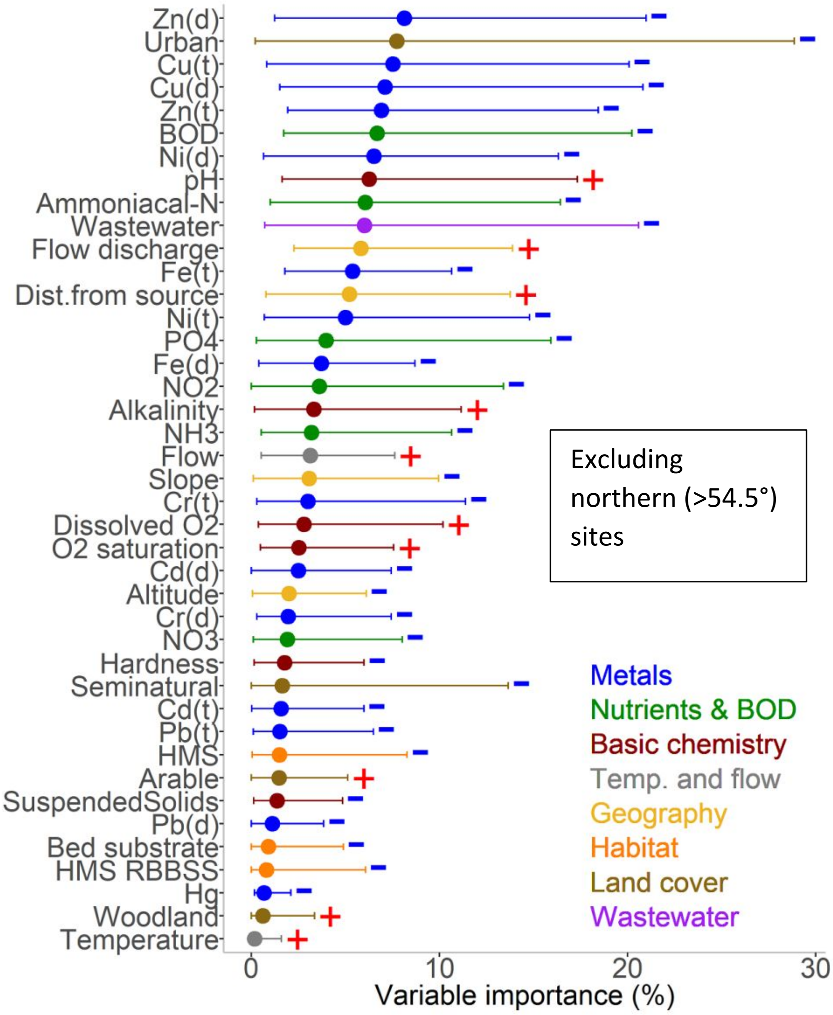

Supporting Figure 6. The relative importance of variables to macroinvertebrate family richness for England based on 1369 sites and 61630 observations restricted to sites below 54.5° latitude (i.e., excluding the Northern Region that is rich in former Zn mining sites) from the GLMM-TMB-NS statistical model, presented as a percentage of explained deviance by each variable. Dots represent mean values across models. Lines indicate the range of explained deviance, with the left and right parts corresponding to minimum and maximum percentages for each variable, respectively. Positive or negative relationships are denoted by plus and minus signs.

Supplementary Materials: R codes

In this analysis, we used the glmmTMB library for fitting Generalized Linear Mixed Models (GLMMs) and the gamm4 library for Generalized Additive Mixed Models (GAMMs).

```
library(glmmTMB)
library(gamm4)
```

GLMM-TMB-NS Models

These models incorporated two chemical variables, one habitat variable, one physical variable, and one land variable as non-linear effects using natural cubic splines (ns), representing a total of five variables of interest. The degrees of freedom (df) for the natural splines were selected based on the Akaike Information Criterion (AIC) for each variable, with a maximum of five degrees of freedom set to prevent overfitting of the model. These models also included an Ornstein-Uhlenbeck time covariance structure (ou), random effects, and seasonal effects.

GLMM-TMB-NS Formulas for the Full Models:

```
formula01 <- as.formula(Family_Richness ~
  #two chemical variables
  ns(chemical_variable1, df=df_cv1)+
  ns(chemical_variable2, df=df_cv2)+
  #one habitat variable
  ns(habitat_variable, df=df_hv)+
  #one physical variable
  ns(physical_variable, df=df_pv)+
  #one land variable
  ns(land_variable, df=df_lv)+
  #seasonal effects
  ns(months, df=df_s)+
  #Ornstein-Uhlenbeck time covariance structure
  ou(time_months + 0 | group)+
  #Random effects
  #We considered and compared two models
  #1. One with nested random effects:
  (1|Region/Site_ID))
  #2. Another model with random effects at the regional level:
  # (1|Region))
```

GAMM models

In the GAMM framework, the number of explanatory variables was reduced to two chemical variables plus one of the following: either a habitat variable, a physical variable, or a land variable, due to convergence issues encountered during model fitting. We employed cubic regression splines (CRS) (s(…,bs = 'cr')) as the smooth functions in our models. CRS were chosen because they effectively capture complex non-linear relationships in the data while helping to prevent overfitting. Additionally, cubic regression splines are computationally more efficient than other spline options, making them suitable for our analysis given the large number of considered models. To model seasonal effects, we used cyclic splines (s(months, bs = 'cc')). We also included a linear year trend effect to account for long-term changes in Family Richness. Due to convergence issues in the more complex models, we opted for this simplified structure to enhance model stability and interpretability.
We recommend ensuring that all variables, including the months (seasonal) variable, are formatted appropriately before conducting the analysis (e.g., as factor variables or numeric values, etc.).

GAMM Formulas for the Full Models:

```
formula02 <- as.formula(Family_Richness ~
  #two chemical variables
  s(chemical_variable1, bs = 'cr')+
  s(chemical_variable2, bs = 'cr')+
  #either a habitat variable, a physical variable, or a land variable
  s(third_variable_of_interest, bs = 'cr')+
  #seasonal effects
  s(months, bs = 'cc')+
  # Linear year trend effect
  year
  # Note: Random effects are not specified in the GAMM formula but are incorporated within the model fitting process as: random = ~ (1|Region/Site_ID).
```

Full Set of Variable Combinations

We generated all possible combinations of predictor variables, representing all potential configurations, as described above. The example below illustrates the process for GLMM-TMB-NS models, where each combination includes two chemical variables, one habitat variable, one physical variable, and one land variable.

For GAMM models, the approach is largely similar; however, instead of looping over habitat, physical, and land variables separately, we combine these three categories into a single category and loop over that combined variable.

```
#example data
chemical_vars <- c("chemical_variable1", "chemical_variable2", "chemical_variable3")
physical_vars <- c("physical_variable1","physical_variable2")
land_vars <- c("land_variable1", "land_variable2")
habitat_vars <- c("habitat_variable1", "habitat_variable2")

#all combinations of two chemical variables
chem_combinations <- combn(chemical_vars, 2, simplify = FALSE)

#all combinations
all_combinations0 <- list()

#loop over each land, physical, and habitat variable
counter <- 1
for (land_var in land_vars) {
  for (physical_var in physical_vars) {
    for (habitat_var in habitat_vars) {

      #loop over each combination of two chemical variables
      for (chem_comb in chem_combinations) {

        #combine the selected variables into one set
        combination <- c(chem_comb, physical_var, land_var, habitat_var)

        #store the combination
```

```
        all_combinations0[[counter]] <- combination
        counter <- counter + 1
      }
    }
  }
}
```

We excluded models that included two versions of approximately the same variable, such as dissolved and total concentrations of a chemical, or related metrics like Ammoniacal Nitrogen and Ammonia, or Dissolved Oxygen and Oxygen Saturation. The following code demonstrates this exclusion process:

```
#pairs to exclude
exclude_pairs <- list(
  c("Ammonia", "AmmoniacalNitrogen"),
  c("OxygenDissolved", "OxygenSaturation"),
  c("CadmiumDissolved", "Cadmium"),
  c("ChromiumDissolved", "Chromium"),
  c("CopperDissolved", "Copper"),
  c("IronDissolved", "Iron"),
  c("NickelDissolved", "Nickel"),
  c("LeadDissolved", "Lead"),
  c("ZincDissolved", "Zinc")
)
should_exclude <- function(combination) {
  any(sapply(exclude_pairs, function(pair) sum(combination %in% pair) > 1))
}
all_combinations <- Filter(function(x) !should_exclude(x), all_combinations0)
```

GLMM-TMB-NS: Comprehensive Analysis

The following code implements a loop that systematically evaluates all possible combinations of predictor variables for GLMMs. For each combination, it first subsets the dataset to include only complete cases, excluding any observations with missing values. The selected variables, excluding the factor variable "DISCHARGE," are then scaled to ensure comparability. Next, a correlation matrix is computed to identify any highly correlated variables, with a threshold set at 0.7; if any pair exceeds this threshold, the loop proceeds to the next combination to avoid multicollinearity issues. A null model is fitted to establish a baseline, and the current combination of variables is extracted for further analysis. The code then generates all possible subsets of the current variable set and constructs both a full model and reduced models for each subset. The deviance for both the null model and the full model is calculated, allowing the assessment of the percentage of explained deviance by the full model. Additionally, the code evaluates the deviance of each reduced model by removing one variable at a time, thereby quantifying the importance of individual predictors:

```
for (ii in 1:length(all_combinations)){
  #subset of complete cases for the current combination of variables
  data0 <- full_data_set[complete.cases(full_data_set[, all_combinations[[ii]]]),]

  #exclude factor variables from scaling and correlation checks
```

```
  vars_to_scale <- setdiff(all_combinations[[ii]], "DISCHARGE")
  data0[, vars_to_scale] <- scale(data0[, vars_to_scale])

  #compute correlation matrix
  ro <- cor(data0[, vars_to_scale])
  #remove diagonal to focus on unique pairs only
  diag(ro) <- NA
  ro <- ro[!is.na(ro)]
  #check if any absolute correlation is above the threshold of 0.7
  #models that include variables with high correlation coefficients are excluded
  if (any(abs(ro)>0.7)){next}

  #Null model
  null0 <- glmmTMB(Family_Richness  ~ 1+(1|Region),
                   data = data0,
                   family=nbinom1(link = "log"))

  #extract the current combination of variables
  comb01 <- all_combinations[[ii]]
  #get the number of variables
  n0 <- length(comb01)
  #generate combinations of the current set of variables
  all_combinations2 <- unlist(lapply((n0-1):n0,function(x) combn(1:n0,x,simplify=F)),recursive=F)

  #full model (the last one) and reduced models
  all.models <- lapply(all_combinations2, function(x) {
    terms <- sapply(comb01[x], function(var) {
      paste0("ns(", var, ", df=", df_var[[var]], ")")  # Access df_var by variable name
    })
    #construct the full formula as a string
    paste0("Family_Richness ~ ",
           paste(terms, collapse = "+"),  #combine all terms
           "+ ns(months, df=df_s) + ou(time_months + 0 | group) + (1 | Region/Site_ID)")
    #alternative model: "+ ns(months, df=df_s) + ou(time_months + 0 | group) + (1 | Region)")
  })

  #Full model
  mod.full <- glmmTMB(as.formula(all.models[[length(all.models)]]),
                      data = data0,
                      family=nbinom1(link = "log"))

  #calculate the deviance of the null model
  deviance.u[ii,'Null']<-
sum(residual_deviance_glmmTMB(data0$Family_Richness,null0,"deviance")^2)
  #calculate the deviance of the full model
  deviance.u[ii,'Full']<-
sum(residual_deviance_glmmTMB(data0$Family_Richness,mod.full,"deviance")^2)
  #calculate the percentage of explained deviance by the full model relative to the null model
```

```
  deviance.u$explained[ii] <- (deviance.u$Null[ii]-deviance.u$Full[ii])*100/deviance.u$Null[ii]

  #calculating deviance for the reduced models
  for (jj in 1:(length(all.models)-1)){
    formula1 <- as.formula(all.models[[jj]])
    #fit the model
    mod1 <- glmmTMB(formula1,data = data0,family=nbinom1(link = "log"))
    #identify the variable removed
    find.var <- comb01[which(c(all_combinations2[[length(all.models)]]) %!in%
c(all_combinations2[[jj]]))]
    #calculate deviance for the current reduced model
    deviance.u2[ii,find.var] <-
      sum(residual_deviance_glmmTMB(data0$Family_Richness ,mod1,"deviance")^2)
    #calculate the percentage of explained deviance by the variable
    deviance.u[ii,find.var] <-
      (deviance.u2[ii,find.var]-deviance.u[ii,'Full'])*100/deviance.u[ii,'Null']
  }
}
```

GAMM: Comprehensive Analysis

The following code implements a loop to evaluate all possible combinations of predictor variables for GAMMs, building on the approach used for GLMMs-TMS-NS. As with the GLMMs, the dataset is subset to include only complete cases, variables are scaled (excluding "DISCHARGE"), and a correlation matrix checks for multicollinearity (threshold of 0.7). A null model is fitted for baseline comparison, and subsets of variables generate full and reduced models. Deviance is calculated for both models to assess explained deviance, and the importance of individual predictors is evaluated by examining the deviance of each reduced model. This maintains consistency with the previously discussed GLMM-TMB-NS framework.

```
for (ii in 1:length(all_combinations)){
  #subset of complete cases for the current combination of variables
  data0 <- full_data_set[complete.cases(full_data_set[, all_combinations[[ii]]]),]

  #exclude factor variables from scaling and correlation checks
  vars_to_scale <- setdiff(all_combinations[[ii]], "DISCHARGE")
  data0[, vars_to_scale] <- scale(data0[, vars_to_scale])

  #compute correlation matrix
  ro <- cor(data0[, vars_to_scale])
  #remove diagonal to focus on unique pairs only
  diag(ro) <- NA
  ro <- ro[!is.na(ro)]
  #check if any absolute correlation is above the threshold of 0.7
  #models that include variables with high correlation coefficients are excluded
  if (any(abs(ro)>0.7)){next}

  #Null model
```

```
 null0<-gamm4(Family_Richness ~ 1,
        random = ~ (1|Region/Site_ID),
        data = data0,
        family=negbin(theta_estimated))

 #extract the current combination of variables
 comb01 <- all_combinations[[ii]]
 #get the number of variables
 n0 <- length(comb01)
 #generate combinations of the current set of variables
 all_combinations2 <- unlist(lapply((n0-1):n0,function(x) combn(1:n0,x,simplify=F)),recursive=F)
 #full model (the last one) and reduced models
 all.models <- lapply(all_combinations2,function(x)paste0("Family_Richness ~",
                              paste0("s(",ColNam02[x],",bs = 'cr')",collapse="+"),
                              "+year+s(months,bs='cc')"))

 #Full model
 mod.full <- gamm4(as.formula(all.models[[length(all.models)]]),
          data = data0,
          random = ~ (1|Region/Site_ID),
          family=negbin(theta_estimated))

 #calculate the deviance of the null model
 deviance.u[ii,'Null']<- sum(residuals(null0$gam, type = "deviance")^2)
 #calculate the deviance of the full model
 deviance.u[ii,'Full']<- sum(residuals(mod.full$gam, type = "deviance")^2)
 #calculate the percentage of explained deviance by the full model relative to the null model
 deviance.u$explained[ii] <- (deviance.u$Null[ii]-deviance.u$Full[ii])*100/deviance.u$Null[ii]

 #calculating deviance for the reduced models
 for (jj in 1:(length(all.models)-1)){
  formula1 <- as.formula(all.models[[jj]])
  #fit the model
  mod1 <- gamm4(as.formula(formula1),data = data0,random = ~
(1|Region/Site_ID),family=negbin(theta_estimated))

  #identify the variable removed
  find.var <- comb01[which(c(all_combinations2[[length(all.models)]]) %!in%
c(all_combinations2[[jj]]))]
  #calculate deviance for the current reduced model
  deviance.u2[ii,find.var] <- sum(residuals(mod1$gam, type = "deviance")^2)

  deviance.u[ii,find.var] <-
   (deviance.u2[ii,find.var]-deviance.u[ii,'Full'])*100/deviance.u[ii,'Null']
 }
}
```

R Code used for the CART analysis

Classification and regression trees (CART) was used by function: *rpart* in R package *party.*

```
#######
# CART #
#######
library(rpart)
library(party)
#Dissolved Zinc
#read database with Zinc and Family richness
FR_Zn_d <-  read_csv("FR_Zn_d.csv")
#Find the critical value of dissolved zinc based on family richness
d.rpart<-rpart(FAMILY_RICHNESS~.,data=FR_Zn_d,control = rpart.control(cp = 0.05))
plot(d.rpart,margin=0.1,main="FR & Zn_dissolved")
text(d.rpart,cex=1)
summary(d.rpart)
printcp(d.rpart)

#Dissolved copper
#read database with copper and family richness
FR_Cu_d <-  read_csv("FR_Cu_d.csv")
#Find the critical value of dissolved copper based on family richness
d.rpart<-rpart(FAMILY_RICHNESS~.,data=FR_Cu_d,control = rpart.control(cp = 0.05))
plot(d.rpart,margin=0.1,main="FR & Cu_dissolved")
text(d.rpart,cex=1)
summary(d.rpart)
```

References

1. Bachiller-Jareno, N. *et al. Macroinvertebrate taxonomic abundance, water quality, river flow, air temperature and environmental site descriptors from English rivers, 1965-2018 [dataset in preparation].* , 2024).
2. Qu, Y. M. *et al.* Significant improvement in freshwater invertebrate biodiversity in all types of English rivers over the past 30 years. *Sci. Total Environ.* **905**, 167144 (2023). https://doi.org/10.1016/j.scitotenv.2023.167144
3. Garcia-Criado, F., Tome, A., Vega, F. J. & Antolin, C. Performance of some diversity and biotic indices in rivers affected by coal mining in northwestern Spain. *Hydrobiologia* **394**, 209-217 (1999). https://doi.org/10.1023/a:1003634228863
4. Kietzka, G. J., Pryke, J. S., Gaigher, R. & Samways, M. J. Applying the umbrella index across aquatic insect taxon sets for freshwater assessment. *Ecological Indicators* **107**, 9 (2019). https://doi.org/10.1016/j.ecolind.2019.105655
5. Moore, R. V., Morris, D. G. & Flavin, R. W. Sub-set of UK digital 1:50,000 scale river centreline network. . (NERC, Institute of Hydrology., Wallingford, UK, 1994).
6. Morris, D. G. & Flavin, R. W. in *Proc 4th International Symposium on Spatial Data Handling.* 250-262.
7. Rowland, C. S. *et al.* Land Cover Map 2015 (vector, GB). (NERC Environmental Information Data Centre, 2017). https://doi.org/https://doi.org/10.5285/6c6c9203-7333-4d96-88ab-78925e7a4e73
8. Williams, R. J. *et al.* A national risk assessment for intersex in fish arising from steroid estrogens. *Environ Toxicol Chem* **28**, 220-230 (2009). https://doi.org/10.1897/08-047.1
9. Jobling, S. *et al.* Predicted exposures to steroid estrogens in UK rivers correlate with widespread sexual disruption in wild fish populations. *Environmental Health Perspectives* **114**, 32-39 (2006). https://doi.org/10.1289/ehp.8050
10. Balaam, J. L. *et al.* The use of modelling to predict levels of estrogens in a river catchment: How does modelled data compare with chemical analysis and in vitro yeast assay results? *Sci. Total Environ.* **408**, 4826-4832 (2010). https://doi.org/10.1016/j.scitotenv.2010.07.019
11. Williams, R. J., Johnson, A. C., Smith, J. J. L. & Kanda, R. Steroid estrogens profiles along river stretches arising from sewage treatment works discharges. *Environmental Science & Technology* **37**, 1744-1750 (2003). https://doi.org/10.1021/es0202107
12. Dawson, F. H., Hornby, D. D. & Hilton, J. A method for the automated extraction of environmental variables to help the classification of rivers in Britain. *Aquat. Conserv.* **12**, 391-403 (2002). https://doi.org/10.1002/aqc.534
13. Robinson, E. L., Blyth, E. M., Clark, D. B., Comyn-Platt, E. & Rudd, A. C. *Climate hydrology and ecology research support system meteorology dataset for Great Britain (1961-2017) [CHESS-met]*, 2020). https://doi.org/10.5285/2ab15bf0-ad08-415c-ba64-831168be7293
14. Raven, P. J. *et al.* River Habitat Survey Quality: the physical character of rivers and streams in the UK and the Isle of Man. . (Environment Agency, Bristol, UK, 1998).
15. Raven, P. J., Holmes, N. T. H., Dawson, F. H. & Everard, M. Quality assessment using River Habitat Survey data. *Aquat. Conserv.* **8**, 477-499 (1998). https://doi.org/10.1002/(sici)1099-0755(199807/08)8:4<477::Aid-aqc299>3.0.Co;2-k
16. Murray-Bligh, J. & Griffiths, M. *Freshwater Biology and Ecology Handbook.* (Foundation for Water Research & Freshwater Biological Association, 2022).
17. Tipping, E., Lofts, S. & Stockdale, A. Metal speciation from stream to open ocean: modelling v. measurement. *Environ. Chem.* **13**, 464-477 (2016). https://doi.org/10.1071/en15111
18. Mebane, C. A. Bioavailability and toxicity models of copper to freshwater life: The state of regulatory science. *Environmental Toxicology and Chemistry* **42**, 2529-2563 (2023). https://doi.org/10.1002/etc.5736
19. Mebane, C. A., Schmidt, T. S., Miller, J. L. & Balistrieri, L. S. Bioaccumulation and toxicity of cadmium, copper, nickel, and zinc and their mixtures to aquatic insect communities. *Environmental Toxicology and Chemistry* **39**, 812-833 (2020). https://doi.org/10.1002/etc.4663

20 Merrington, G. *et al.* Deriving a bioavailability-based zinc environmental quality standard for France. *Environ. Sci. Pollut. Res.* **28**, 1789-1800 (2021). https://doi.org/10.1007/s11356-020-10603-8

21 Peters, A. *et al.* Evaluating the protectiveness of a bioavailability-based environmental quality standard for the protection of aquatic communities from zinc toxicity based field evidence. *Environmental Toxicology and Chemistry* **42**, 1010-1021 (2023). https://doi.org/10.1002/etc.5570

22 Joachim, S. *et al.* A long-term copper exposure in a freshwater ecosystem using lotic mesocosms: Invertebrate community responses. *Environ Toxicol Chem* **36**, 2698-2714 (2017). https://doi.org/10.1002/etc.3822

23 Wood, S. N. *Generalized Additive Models: An Introduction with R*. 2. edn, (Chapman and Hall/CRC Press, 2017).

24 Wood, S. N. gamm4: Generalized additive mixed models using mgcv and lme4 v. R package version 0.2-6 (2022).

25 Oedekoven, C. S. *et al.* Attributing changes in the distribution of species abundance to weather variables using the example of British breeding birds. *Methods Ecol. Evol.* **8**, 1690-1702 (2017). https://doi.org/10.1111/2041-210x.12811
